# Cooperative folding as a molecular switch in an evolved antibody binder

**DOI:** 10.1016/j.jbc.2024.107795

**Published:** 2024-09-19

**Authors:** Malin Jönsson, Ameeq Ul Mushtaq, Tamás Milán Nagy, Emma von Witting, John Löfblom, Kwangho Nam, Magnus Wolf-Watz, Sophia Hober

**Affiliations:** 1 Department of Protein Science, KTH-Royal Institute of Technology, Stockholm, Sweden; 2 Department of Chemistry, Umeå University, Umeå, Sweden; 3 Department of Chemistry and Biochemistry, University of Texas at Arlington, Arlington, Texas, USA

**Keywords:** protein switch, protein engineering, directed evolution, allostery, metal-dependency, calcium, NMR

## Abstract

Designing proteins with tunable activities from easily accessible external cues remains a biotechnological challenge. Here, we set out to create a small antibody-binding domain equipped with a molecular switch inspired by the allosteric response to calcium seen in naturally derived proteins like calmodulin. We have focused on one of the three domains of Protein G that show inherent affinity to antibodies. By combining a semi-rational protein design with directed evolution, we engineered novel variants containing a calcium-binding loop rendering the inherent antibody affinity calcium-dependent. The evolved variants resulted from a designed selection strategy subjecting them to negative and positive selection pressures focused on conditional antibody binding. Hence, these variants contains molecular “on/off” switches, controlling the target affinity towards antibody fragments simply by the presence or absence of calcium. From NMR spectroscopy we found that the molecular mechanism underlying the evolved switching behavior was a coupled calcium-binding and folding event where the target binding surface was intact and functional only in the presence of bound calcium. Notably, it was observed that the response to the employed selection pressures gave rise to the evolution of a cooperative folding mechanism. This observation illustrates why the cooperative folding reaction is an effective solution seen repeatedly in the natural evolution of fine-tuned macromolecular recognition. Engineering binding moieties to confer conditional target interaction has great potential due to the exquisite interaction control that is tunable to application requirements. Improved understanding of the molecular mechanisms behind regulated interactions is crucial to unlock how to engineer switchable proteins useful in a variety of biotechnological applications.

Essential to the complexity of regulation found in nature are allosteric proteins which upon a specific stimulus can change conformation leading to a responding action (1, 2, 3). The input stimulus could for instance be a binding event of a small molecule or ion, a physical change in pH, or light exposure leading to a functional response such as the interaction with a ligand, oligomerization, or enzyme activity (4). The interest in engineering novel protein switches inspired by the natural repertoire is fueled by the extensive applicability, including screening biomarkers for different diseases, detecting environmental toxins, developing highly sensitive therapies triggered in a certain environment, and enabling selective therapeutics such as prodrug-activating enzymes (5).

Novel domains, where the protein’s function can be regulated, could be developed by engineering naturally existing allosteric proteins or by combining protein domains with various inherent properties to generate a sought-after function. Both approaches pose great design challenges which are often eased by using combinatorial libraries to create protein diversity to which directed evolution or high-throughput screens can be applied to identify the rare variants with desired switch-like properties. Examples include successful engineering efforts where β-lactamase was inserted into sugar-binding proteins to render its activity regulated by the sugar (6) or where a metal-binding site was grafted onto bacterially derived protein domains with an inherent affinity for antibodies to control this affinity through the supply or removal of metal ions (7, 8).

Bacteria such as staphylococci and streptococci have evolved to express surface proteins with an inherent affinity to immunoglobulin G (IgG) with broad species specificity as a means to evade the immune response (9). As proven through several engineering efforts (7, 8, 10), these proteins can serve as scaffolds for engineering since they are well characterized in terms of structural properties, interactions, and thermodynamic stability (11, 12, 13). One of these IgG-binding proteins is streptococcal Protein G, which consists of small domains that can bind either serum albumin or IgG originating from various mammalian species. NMR and crystallography studies have shown that a single antibody-binding domain denoted C2, consisting of four anti-parallel beta-sheets crossed over by an alpha helix can interact with both the Fc- and Fab-region of IgG (12). However, the affinities reported for these binding sites vary depending on species, attributed to structural differences; for example, the predominant interaction with mouse IgG1 occurs with the Fab region (13) while for all human IgG subclasses the affinity to the Fab-region is only about 10% of the affinity to Fc (12).

Here, we present the development of a semi-rationally designed metal-dependent Protein G domain. Introduction of a randomized calcium-binding loop into the naturally occurring antibody-binding protein domain combined with directed evolution using bacterial display resulted in several designed proteins comprising switch-like binding properties with intact affinity to human and mouse IgG solely in the presence of calcium. NMR and molecular dynamics (MD)-based structural analysis of the engineered domain in comparison to the wild type, both in the presence and absence of calcium, revealed insights into the molecular mechanisms underlying the evolved calcium dependency. Collectively, our findings suggest that the adaptation of the system to function under the exerted selective pressure was a coupled folding and calcium-binding reaction that resulted in intact target-binding interfaces only in the presence of bound calcium.

## Results

Here we present an approach to develop a molecular switch and at the same time provide the underlying molecular mechanism of the switching capacity. The concept is to replace an existing short loop of the C2 protein with a variable loop containing a composition inspired by EF-hand motifs. By employing negative and positive selective pressures in terms of antibody-binding activity in the absence (negative pressure) and presence (positive pressure) of calcium we search for tunable variants in a high-throughput setup. For variants successfully displaying calcium-dependent antibody affinity, we then determine the underlying molecular mechanism of the calcium response to develop a conceptual advance that can be broadly transferred to other systems.

### Design of a calcium-coordinating library

**Figure 1:**
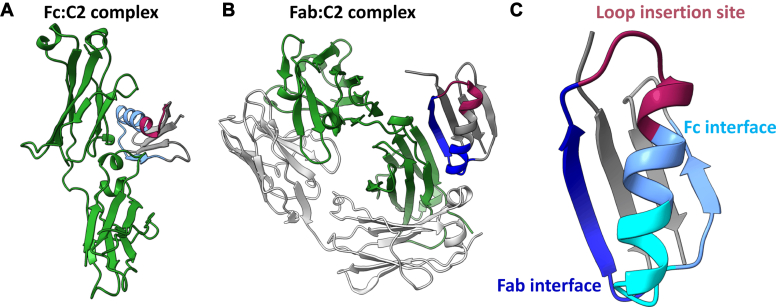
**Structural basis for engineering of a calcium-binding loop in relation to inherent target interaction sites.** In (*A*–*C*) the location of the loop in C2 that is replaced with a prospect calcium-binding sequence is colored in burgundy. *A*, crystal structure (PDB: 1FCC (12)) showing the interaction between human Fc and C2. The Fc interaction interface on C2 is colored in light blue. *B*, crystal structure (PDB: 1IGC (14)) of the C2 domain interacting with a human Fab fragment. The Fab interaction surface on C2 is colored in dark blue. *C*, ribbon structure of C2 (PDB: 1FCC) highlighting the antibody-binding sites of Fab (*dark blue*) and Fc (*light blue*) in the parental C2 domain as well as the chosen insertion site for the metal-coordinating loop library (*burgundy*). The Fab and Fc interaction interfaces are partially overlapping and this is shown in cyan (12).

The protein structure of C2 including its previously characterized antibody-binding surfaces was analyzed to find a suitable insertion site for a metal-coordinating loop that could render the inherent target affinity calcium-dependent. As inferred from the crystal structures (PDB: 1FCC (12) and 1IGC (14)), the binding surface for Fc is at the C-terminal end of the alpha helix, extending into the third beta-sheet (Fig. 1*A*), while the Fab binding surface is mainly located to the second beta-sheet and the C-terminal end of the alpha helix (Fig. 1*B*). Hence, it was hypothesized that grafting of a calcium-binding loop preceding the alpha helix might affect both binding regions and thereby infer a calcium dependency without risking the complete loss of structural integrity and functionality (Fig. 1*C*). Measures to further prevent disrupting the protein structure were taken by including the option of a flexible linker N-terminally of the loop. Therefore, the library was designed to include four different linker lengths of 0 to 3 amino acids. The three possible linker positions were designed with an equimolar mix of serine, threonine, glycine, and proline (Fig. S1) which were chosen to allow for different characteristics such as polarity (Ser/Thr), flexibility (Gly), and rigidity (Pro). Following the linker, the library was intended to cover a large number of possible calcium-binding loops consisting of 13 amino acids with designed randomizations in all but two positions which were found throughout literature to be evolutionarily conserved in most known EF-hand motifs (Fig. S1) (15, 16).

### Enrichment of calcium dependency throughout the selection process

**Figure 2:**
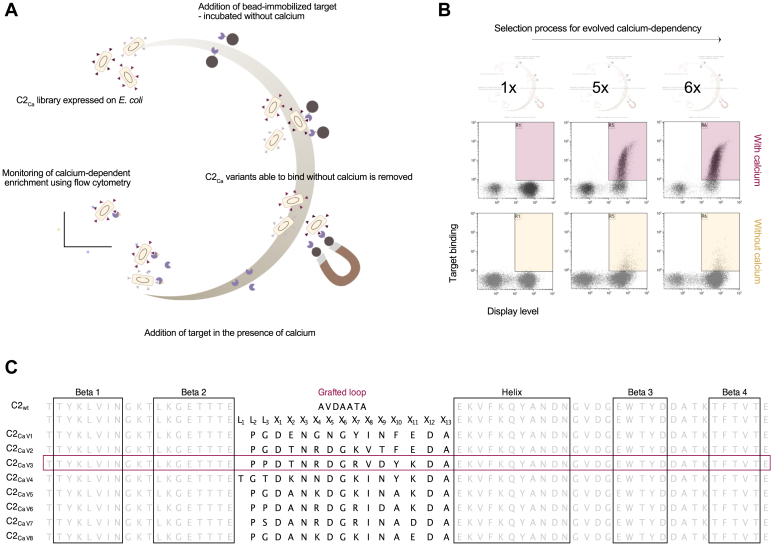
**Overview of the directed evolution approach designed with negative and positive selection pressures.***A*, selection process for directed evolution of calcium-dependent protein domains using MACS and FACS. *B*, dot plots of the *E. coli*-displayed library showing enrichment of calcium-dependent target interaction throughout the selection process. Fluorescence intensity (FI) corresponding to the display level is shown on the X-axis, and FI corresponding to the target (mIgG1) binding is shown on the Y-axis for the first (1×) and last two rounds (5× and 6×) of selection. Representative gates are illustrated to ease the comparison of target interaction in the presence (*burgundy*) and absence of calcium (*yellow*) throughout the rounds. *C*, sequence comparison of the parental domain C2_wt_ and the eight characterized library variants with the representative variant C2_Ca V3_ highlighted in *burgundy*. The inserted library consists of a flexible linker (L_1-3_) preceding the designed calcium-binding loop (X_1-13_).

Directed evolution was implemented to isolate calcium-dependent versions of C2 by displaying the designed library on *Escherichia coli* cells, and performing an iterative cycle of negative and positive selection (Fig. 2*A*). The negative selection consisted of using magnetic-activated cell sorting (MACS) by immobilizing the target on magnetic beads and incubating beads with cells in a buffer containing a chelator to ensure a calcium-free environment to remove variants that could interact with the target in the absence of calcium. Variants with undesired characteristics, *that is,* binding to the target-immobilized beads calcium independently, were removed by magnetic capture while the variants that remained in solution were subsequently exposed to the same target but this time in a buffer supplied with calcium during a positive selection. Displaying the library on cells enabled the advantage of high-throughput analysis using fluorescence-activated cell sorting (FACS). The display method includes a reporter protein consisting of an albumin-binding domain expressed alongside the autotransporter used to localize and anchor the protein library to the cell surface. Therefore, library expression levels can be simultaneously monitored in real-time through a differently labeled fluorophore (human serum albumin-Alexa647 conjugate) than the fluorophore used to detect target binding (Streptavidin R-Phycoerythrin, SAPE), imparting a means to normalize signals obtained for target interaction against the number of displayed proteins (17). FACS was employed during the positive selection step to isolate variants that expressed the library (X-axis) and simultaneously showed target binding (Y-axis) from the non-expressing or non-binding cell populations. Additionally, continuous monitoring of the selection process was done using flow cytometry, and this revealed enrichment of calcium-dependent clones throughout the iterative rounds (Fig. 2*B*).

After six rounds, 192 clones were isolated and screened using flow cytometry for calcium-dependent target binding. From the 192 screened variants, 132 clones did display promising characteristics, while 60 clones showed target interaction independently of the presence of calcium and were discarded. All 132 conditional binders were sent for Sanger sequencing, and these appeared as unique but with sufficient similarities to perform a clustering alignment from which eight variants, one from each resulting cluster, were chosen for further characterization (Fig. 2*C*). The selected variants were produced and purified to homogeneity as confirmed by mass spectrometry, SDS-PAGE, and size exclusion chromatography (SEC, Fig. S2). C2_Ca V3_ is the representative example displayed from here on.

### Kinetic evaluations show calcium-dependent antibody interactions for the selected variants

**Figure 3:**
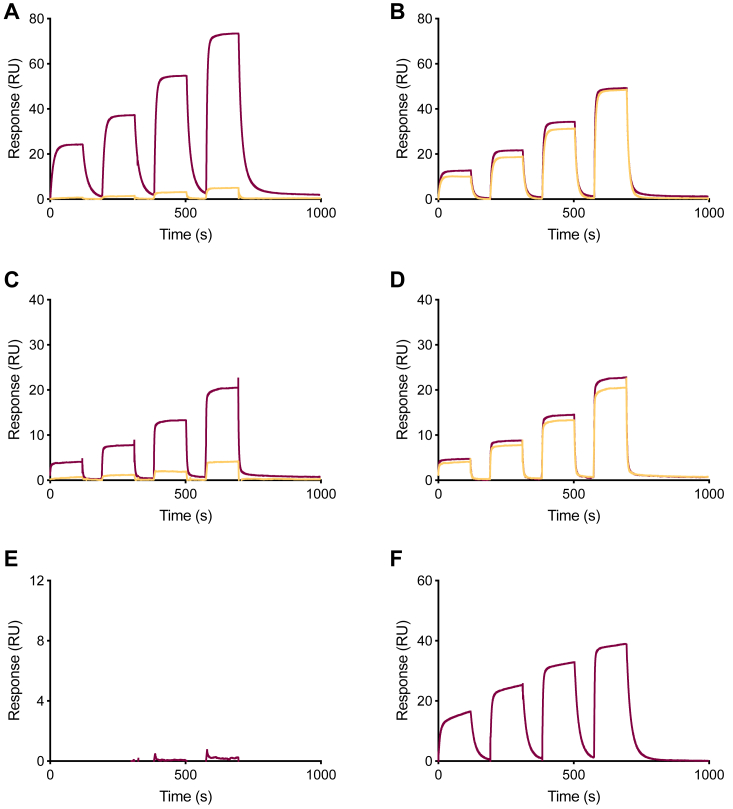
**Antibody interaction is switchable and calcium-dependent for the evolved C2 variants.** Measured target interaction, in the presence (*burgundy*) and absence (*yellow*) of calcium, for the representative example C2_Ca V3_ (*left panel*) and the original C2_wt_ (*right panel*). Both variants were injected over an SPR-surface immobilized with either mouse IgG1 (*A* and *B*), human Fab (*C* and *D*), or human Fc (*E* and *F*) in a concentration series (250 nM – 2000 nM). While the interaction between C2_Ca V3_ and mIgG1 as well as hFab was detectable only in the presence of calcium, the control (C2_wt_) displayed a calcium-independent target association. Further, C2_Ca V3_ has lost its inherent binding to hFc and no interaction was detected in the presence of calcium compared to the control (C2_wt_).

The selective pressure from the directed evolution was designed to evolve C2 variants that bound target antibodies only in the presence of calcium. Whether the inherent antibody interaction had truly been rendered calcium-dependent was assessed by surface plasmon resonance (SPR). Full-length monoclonal mouse IgG1 was immobilized and a concentration series of the evolved variants, as well as the wild type, was injected in either a calcium-containing buffer or a buffer containing a chelator to ensure a calcium-free environment. The interaction of the evolved variants was benchmarked against C2_wt_ and showed similar kinetic parameters towards mIgG1 in the presence of calcium (Table S1). However, for all evolved variants this target interaction was switched off in the absence of calcium while, as anticipated, the affinity of C2_wt_ was not significantly affected. Here, the change in response is within the experimental uncertainty (https://cdn.cytivalifesciences.com/api/public/content/digi-33041-pdf) (Figs. 3, *A* and *B* and S3). Hence, the applied selection pressure resulted in the engineering of a calcium-dependent switching feature. Moving further to analysis of binding towards polyclonal human Fab, using a similar experimental set-up, the calcium-dependent interaction was re-iterated for all evolved variants displaying fast binding in the presence of calcium, comparable to the wild type, and a target association below the limit of detection in the absence of calcium (Figs. 3, *C* and *D* and S4). Surprisingly, although the wild type shows affinity for human Fc (Fig. 3*E*, Table S1), no binding could be detected between the representative example C2_Ca V3_ and hFc neither in the absence nor the presence of calcium (Fig. 3*F*). As was the case for a majority of the evolved variants (Figs. S5-S7). These results suggest that the molecular switch that evolved in response to the applied selection pressure has its origin in the interaction with the Fab moiety of the antibody. Taken together, the kinetic values demonstrated that the evolved C2 variants harbor functional and calcium-dependent molecular switches. Furthermore, it appears likely that the variants adapted to the selective pressure promoting the Fab-binding at the expense of interaction with the Fc moiety.

### Calcium-dependent stability and secondary structure

**Figure 4:**
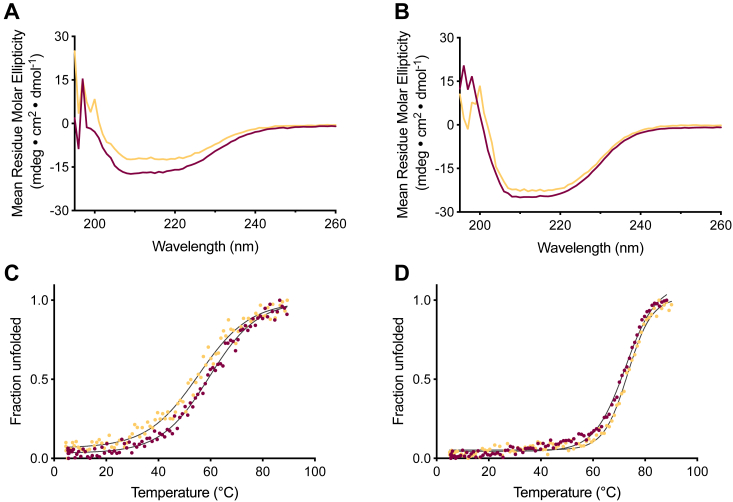
**The evolved variants in comparison to C2**_**wt**_**indicate a structural calcium dependency.** The structural integrity and thermal stability of the selected variants was investigated with CD spectroscopy. Experiments with and without calcium are colored in *burgundy* and *yellow*, respectively. Observation of secondary structure elements of C2_Ca V3_ (*A*) and the parental C2_wt_ domain (*B*) was performed with far-UV CD spectrum. Assessment of thermal stability through quantification of fraction unfolded protein of the CD signal at 221 nm in response to temperature for (*C*) C2_Ca V3_, and (*D*) the parental C2 domain. The solid lines represent a fit to a two-state folding/unfolding model. The *T*_m_ values in the absence/presence of calcium was 56/62 °C for C2_Ca V3_ and 75/75 °C for the parental C2 domain, respectively.

Previous studies have shown that calcium-binding loops can undergo conformational changes upon binding of calcium causing them to become less flexible and that change can be reflected in the secondary structure and thermal stability of the entire protein (7, 18). Thus, the structural integrity of the variants was analyzed with far-UV circular dichroism (CD) spectra that are sensitive to secondary structure content, and the thermal stability was quantified by monitoring the temperature dependency of the CD signal at 221 nm. In Figure 4 the data for the C2_Ca V3_ variant is shown, chosen as a representative example, while the data for the remaining variants is displayed in Figures S8 and S9. By comparing the CD spectrum of C2_Ca V3_ (Fig. 4*A*) against that of the wild type protein (Fig. 4*B*) in both the presence and absence of calcium, there are two features that are particularly noteworthy. First, the spectrum of C2_Ca V3_ in the absence of calcium demonstrates that the protein indeed contains secondary structure, and second, the secondary structure content is increased in the presence of calcium which indicates a direct physical interaction between the variant and calcium. The thermal unfolding of C2_Ca V3_ occurs with a cooperative transition both in the presence and absence of calcium, demonstrating that the variant is a folded protein in both conditions (Fig. 4*C*). Although the transition is cooperative it occurs over a broader temperature interval compared to the wild type (Fig. 4*D*) which is fully consistent with the fitted enthalpies of unfolding (130 and 236 kJ mol^-1^ for C2_Ca V3_ and C2_wt_ in the absence of calcium, respectively). The most likely effect of a perturbation of a protein structure is reduced stability (19), however, the melting points (*T*_m_) of C2_Ca V3_ is 56 °C (without calcium) and 62 °C (with calcium) which is well above the threshold required for further structural analysis. Taken together, the data demonstrates that the selected variants are folded proteins with sufficiently high thermal stabilities and that the effect of calcium reflected in the function is likely an ordering of the grafted calcium-binding loop and potentially also residues before and after the loop. In search for the underlying molecular mechanism of the evolved interaction pattern, we turned to liquid-state NMR spectroscopy as described in the following section.

### Molecular mechanism of the evolved calcium switch from NMR and MD

The analysis of antibody-binding, structure, and stability of the evolved variants clearly demonstrated that a calcium-dependent molecular switch was generated and that the designed variants, although predominantly folded proteins, experienced a gain in secondary structure upon calcium-binding. Therefore, the molecular mechanism of the calcium switch is likely linked to an ordering event in response to calcium. To decode the mechanism at the level of individual amino acid residues, NMR spectroscopy, which is uniquely suited to provide mechanistic understanding at this level, was utilized.

**Figure 5:**
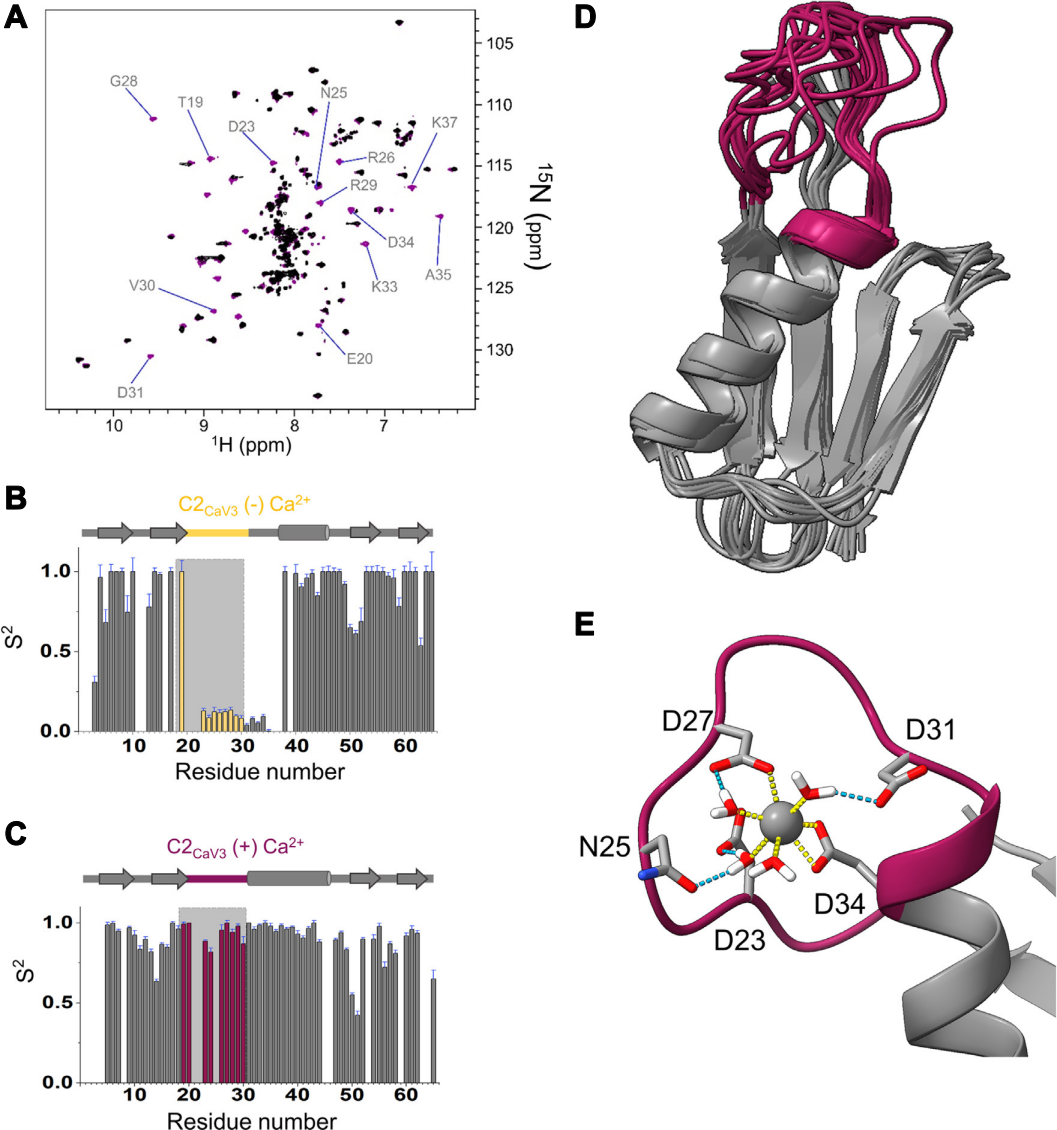
**NMR evidence for ordering of C2**_**Ca V3**_**in the presence of calcium.** Solution-state NMR spectroscopy was used to determine the mechanism underlying the calcium-dependent binding to antibodies through the determination of the structure and dynamics in response to calcium binding. *A*, overlay of ^1^H-^15^N HSQC spectra of 0.4 mM C2_Ca V3_ without calcium present (*black*) and 0.4 mM C2_Ca V3_ with 3.2 mM calcium added (*burgundy*). Assignment of residues in the calcium-binding loop of C2_Ca V3_ in the presence of bound calcium is shown with labels. *B and C*, quantification of dynamics for backbone amide (^15^N-^1^H) order parameters (S^2^) along the residue numbers for (*B*) C2_Ca V3_ in the absence of calcium and (*C*) C2_Ca V3_ in the presence of calcium. The residues of the grafted loop in C2_Ca V3_ are highlighted in *yellow* in the absence of calcium (*B*), and in *burgundy*-colored bars for the presence of calcium (*C*). The root-mean-square errors associated with each S^2^ value are shown in each bar graph (*blue*). All spectra were measured at 850 MHz field in 20 mM MES buffer at pH 6.0 and 20 °C. *D*, NMR-derived solution structure (*i.e.* the ten lowest energy CS-Rosetta models) of C2_Ca V3_ in the presence of calcium with the grafted loop highlighted in *burgundy*. *E*, coordination of calcium from a 1 μs MD simulation. The octahedral calcium coordination geometry is indicated with dashed *yellow lines* and hydrogen bonds to coordinating water molecules are shown as dashed *blue lines*.

A comparison of assigned ^1^H-^15^N HSQC spectra of C2_wt_ and C2_Ca V3_ (Fig. S10) reveals that, although C2_Ca V3_ is a globally folded protein, resonances corresponding to the evolved calcium-binding loop are located in the spectral region expected for disordered residues. Therefore, it appears that the grafted calcium-binding loop is disordered in the absence of calcium while the remainder of the protein is well folded. The addition of calcium to C2_Ca V3_ resulted in a significant increase in the dispersion of resonances that correspond to residues in the calcium-binding loop relative to the calcium-free case (Figs. 5*A* and S11). These chemical shift changes suggest that calcium-binding to C2_Ca V3_ occurs with a coupled folding and binding mechanism where residues in the calcium loop undergo a disorder-to-order transition upon calcium coordination.

To evaluate the coupled folding and binding model from the standpoint of protein dynamics, we quantified fast ps-ns dynamics of C2_Ca V3_ in the absence and presence of calcium from NMR spin relaxation including ^15^N-{^1^H}-heteronuclear nuclear Overhauser effects (hnNOEs), R_1_ and R_2_ relaxation rates (Fig. S12). The data were interpreted by order parameters using the model-free approach (20). In the calcium-free case, the calcium-binding loop and the flanking residues of the loop are clearly disordered based on their low-order parameters (Fig. 5*B*). Notably the disordered segment encompasses the C-terminus of the Fab-binding surface and the N-terminal part of the Fc-binding surface. The situation changes dramatically when calcium is bound as evidenced by increased order parameters of the calcium-binding loop that now match the values of the structured regions of the protein (Fig. 5*C*). The NMR-derived fast time-scale dynamics thus reinforce the conclusions from the chemical shift perturbations that the grafted loop is disordered in the absence, but well-ordered in the presence of calcium, and that C2_Ca V3_ interacts with calcium with a coupled folding and binding mechanism. Similar observations of ordering based on fast time-scale dynamics have been made for other cases that are rooted in coupled folding and binding mechanisms (21).

To gain structural insights into the calcium coordination by C2_Ca V3_, a solution structural model was determined with CS-Rosetta (22). The CS-Rosetta structure of C2_Ca V3_ in the presence of calcium was generated from assigned backbone chemical shifts and the ten lowest energy structures are shown in Figure 5*D* (PDB ID: 9FA8). The structure has the expected overall C2 topology but with an extension corresponding to the designed calcium-binding loop. Since calcium is silent to NMR spectroscopy detected on ^1^H, ^15^N, and ^13^C nuclei, we performed a 1 μs MD simulation based on the lowest energy structure in order to define the contacts that are responsible for calcium coordination (Fig. 5*E*). In C2_Ca V3_, calcium exhibits octahedral coordination, with direct (or inner shell) sidechain interactions to calcium from residues Asp23, and Asp27 and a bi-dentate interaction from Asp34. Water-mediated interactions (outer shell interactions) are made from residues Asp23, Asn25, Asp27, and Asp31 (Fig. 5*E*). Among the interactions, the MD simulation shows that Asp34 consistently coordinates with calcium, while three Asp residues (*i.e.*, Asp23, 27, and 31) exchange coordination, with only one residue forming direct coordination with calcium at any given time, with Asp23 forming the longest-lasting coordination with calcium (Fig. S13).

A distinct molecular explanation for the calcium-dependent binding towards hFab emerges from the NMR data. While chemical shifts and dynamics demonstrate that the binding surface towards hFab (and hFc) is partially disrupted in the absence of calcium, the NMR data show that the binding surfaces are fully restored in the presence of calcium. Therefore, the nature of the molecular mechanism that controls the calcium-dependent binding of hFab can be classified as “coupled folding and binding”. The underlying microscopic event that drives the observed macroscopic coupled folding and binding, may be conformational selection (20) where calcium binds to loop orientations that have sampled bound-like structures. The selection experiment was designed against hFab and as a side-effect the evolved C2_Ca V3_ variant had lost its hFc binding capacity. A plausible explanation for the loss of hFc binding emerges from a superimposition of the C2_Ca V3_ NMR structure on the C2 protein in the C2:hFc complex (PDB: 1FCC (12)). In this superimposition, the backbone of residues in the vicinity of Thr24 and Tyr32 of calcium-bound C2_Ca V3_ makes severe clashes with atoms in the hFc (Fig. S14), and this interference then likely acts to disrupt the formation of a C2_Ca V3_: hFc complex. However, there exist selected variants that retain their binding capacity towards hFc (Fig. S5, *F* and *G*) and these variants then lack the steric clashes that interfere with hFc binding by C2_Ca V3_.

The direct molecular interaction between C2_Ca V3_ and hFab was confirmed by ^15^N-HSQC chemical shift mapping of the C2_Ca V3_ resonances in a hFab complex in the presence of calcium (Fig. S15). Uniform peak broadenings were observed for C2_Ca V3_ resonances without significant chemical shift perturbations. This behavior indicates strong binding on the NMR scale and an intermediate exchange rate between the free and bound forms. Since the signal attenuation is uniform for the C2_Ca V3_ resonances, no distinct hFab binding surface on C2_Ca V3_ could be identified. Further dynamical analysis of the complex would provide insights into the binding mechanism. However, since around half of the resonances are either near the detection limit or broadened beyond detection, specific studies of the complex are not feasible without advanced labeling strategies.

## Discussion

Naturally occurring proteins have evolved to incorporate and transmit signals or regulate their level of activity through allosteric and induced conformational changes in the protein structure. The introduction of switchable proteins responding to easily accessible external cues has significant potential in biotechnological and medical applications, such as for specific activation under defined disease conditions or, related to the present findings, purification of medically relevant antibodies (7, 8, 23, 24). In addition, mechanistic insight into the underlying molecular properties of an evolved switch provides knowledge that, on a general level, can be used in the design of proteins with novel properties (25).

By constructing a combinatorial library with a calcium-binding loop introduced at a strategic location in the antibody-binding protein C2 and displaying this on cells it was possible to efficiently screen the protein library members for intact structures on the basis that those could be expressed and interact with the native antibody target. Exposure of the variants to negative and positive selective pressures enabled enrichment of variants that were shown to contain antibody affinities which were conditionally controlled by the presence of calcium. Although the evolved variant that we focus on here (*i.e.* C2_Ca V3_) displayed a reversible interaction towards full-length IgG1 it had lost its affinity for hFc. Thus, the binding towards the full-length antibody is conferred to the Fab part. These binding properties highlight a fundamental aspect of evolution, namely that a system that evolves against a selective pressure develops features that will generate an evolutionary advantage towards the applied pressure in question. In light of our observations, this shows that a calcium-dependent affinity towards a full-length antibody can evolve by retaining the binding to either Fab or Fc moieties or, in theory, to both moieties depending on the selection pressures applied.

Similarly, sequencing of the calcium-dependent variants showed that the positively charged amino acid (K) was dominant in position 11 which is also commonly seen in known EF-hand motifs like the calmodulin loop sequence (DKDGDGTITTKE). Another common feature with naturally evolved calcium-binding is the abundance of negatively charged/polar residues (D/N) throughout the grafted loop, and these residues were indeed, from our structural analysis, shown to be vital for calcium coordination. Interestingly, the modeling results also revealed a consistent coordination of calcium from Asp34 in a bidentate fashion, as commonly seen for Glu in this position, which might improve selectivity for calcium over magnesium according to literature since Asp interacts monodentate with Mg^2+^ (15, 16, 26).

With the evolved variants, and in particular C2_Ca V3_, we have discovered a robust system for which we could decode the underlying molecular mechanism for the evolved and switchable antibody affinities. For NMR experiments it was found that the calcium-dependent molecular switch was controlled with a “coupled folding and binding” transition. The “off state” that is dominating in the absence of calcium is partially disordered, with a disrupted Fab-binding surface and an unfolded calcium-binding loop. In contrast, the “on state” that is present only in the presence of calcium is fully ordered and with a restored Fab-binding surface. The principle of coupled folding and binding that evolved under the selective pressure exerted in this study is, in essence, identical to the same principle that has evolved over and over again in nature. Notable examples of naturally evolved “coupled folding and binding” in response to target protein interaction include the activation domain of herpex simplex virus protein VP16 (27), the chaperone binding domain of the *Yersinia* effector protein YopE (21), and the pKID domain of the nuclear factor CREB (28).

The solution structure of C2_Ca V3_ determined in the presence of calcium provided an explanation for the loss of Fc-interaction. This effect is due to a steric clash between Tyr32 of the evolved variant with a serine in the Fc-part of the antibody. Taken together, the NMR and MD analysis provided the mechanism of both the evolved calcium switching function and of the loss of Fc-binding. A key feature in this design concept is that the engineered calcium-binding loop is flanked by the Fab- and Fc-binding surface. Consequently, the same design principle can be used also for other systems that meet such requirements.

## Conclusion

An elegant way to control protein activity is by creating a functional change through the alteration of a protein’s tertiary structure. Here, we engineered a calcium-binding loop into a small domain of streptococcal Protein G and evolved calcium-dependency in the sense that upon binding of this ion, these domains switch from an inactive state to a functional one rending the inherent target affinity to antibodies and antibody fragments allosterically controlled. Once depleted of calcium, the target interaction of these proteins is lost. Structural analysis explained the molecular mechanism that evolved from the selection pressures and revealed that the introduced calcium dependency was linking cooperative protein folding of the grafted loop with switchable target binding. Designing binding moieties to confer calcium dependency is expanding the field of protein engineering, enabling the development of protein switches where the desired target affinity could be tailored to be turned on or off based on application. This proof-of-concept study could serve as a model system for engineering molecular switches applicable in a variety of biotechnological settings such as in the creation of selective protein therapeutics or the development of milder antibody purification procedures. Furthermore, this cross-disciplinary study of engineered allosteric proteins might lay the foundation for exploring theories on how allostery evolves.

## Experimental procedures

### Library creation

The library was designed to consist of the C2 domain with a randomized stretch of 13 amino acids between the second beta strand and the alpha helix (Fig. S1). Upstream of this stretch, a linker region of 0 to 3 amino acids was included and oligonucleotides encoding this library were synthesized and NGS-verified (Twist Bioscience). 200 ng of DNA was PCR-amplified during 20 cycles before performing traditional cloning to insert it into the pBad2.2 *E. coli* display vector (17). The vector is designed with an expression cassette including the genes encoding the library followed by a gene encoding an engineered albumin-binding domain which is used as a reporter tag for monitoring display level. Expression is under the control of a titratable arabinose promoter (17). The final ligation product was electroporated into BL21(DE3) cells (Lucigen) which were cultivated in 100 ml Luria Bertani (LB) media supplemented with 100 μg/ml carbenicillin overnight at 37 °C, 150 rpm. Cells were harvested through centrifugation the following morning and the practical library size was calculated to be 1.5 × 10^8^ variants through replicate titrations. Stocks containing 15% glycerol were made with 1.5 × 10^9^ cells and stored at −80 °C.

### Target preparation

Mouse IgG1 (mIgG1, RRID: AB_2339005) and human polyclonal Fab fragment (hFab, RRID: AB_2337045) were ordered from Jackson ImmunoResearch and biotinylated using EZ-link Sulfo-NHS-LC-Biotin (Thermo Scientific) according to the manufacturer’s instructions. The biotinylation was stopped by removing non-reacted biotin using a NAP5 desalting column (Cytiva).

### *Escherichia coli* display selections

1.5 × 10^9^ cells were cultivated in 100 ml LB containing 100 μg/ml carbenicillin at 37 °C, 150 rpm until the OD reached 0.5 to 0.8 and the culture was induced with 0.6% w/v L-arabinose and grown overnight at 25 °C. The induced *E. coli* cells were washed with TBSC (50 mM Tris, 150 mM NaCl, 1 mM CaCl_2_, pH 7) before being resuspended in the same buffer but containing 150 nM of biotinylated mIgG1 or 500 nM of biotinylated hFab and incubated on a rotamixer at room temperature (RT) for 2 hours. Next, the cells were washed with TBSC, resuspended in 150 nM human serum albumin (HSA)-Alexa 647 conjugate (in-house produced), and 2 μg/ml streptavidin conjugated with R-phycoerythrin (SAPE) (Invitrogen) and incubated dark on ice for 30 min. The cells were subsequently washed and lastly resuspended in cold TBSC before being sorted using a MoFlo Astrios EQ cell sorter (Beckman Coulter). The sort gate was set to capture cells displaying the library (detected library expression with (HSA)-Alexa-647 conjugate, X-axis) and binding to the target (detected target binding with SAPE, Y-axis). Sorted cells were collected in a 15 ml tube containing LB media and were incubated during rotation for 1 h at 37 °C before being inoculated to 150 ml of LB media supplemented with 100 μg/ml carbenicillin and cultivated overnight at 37 °C, 150 rpm. Six rounds of selection in solution were performed following this procedure, round 2 to 6 also included a negative selection step using MACS prior to the positive FACS described. MACS was implemented to remove non-calcium-dependent binders by pre-incubating target (150 nM mIgG1 or 500 nM hFab) with streptavidin-coated Dynabeads MyOne Streptavidin C1 (Thermo Scientific) before allowing the cells to be incubated with the target-coated beads for 2 h in MBSE (25 mM MES, 150 mM NaCl, 100 mM EDTA, pH 5.5). The magnetic beads were thoroughly washed and captured and the resulting supernatant was used as input for FACS.

### Flow cytometry screening of selection output

The selection output was screened for target interaction in buffer either containing calcium or a chelator (EDTA) using the following procedure, initially as pools of sorted clones after each selection round and then as single colonies obtained from the final selection round. The single colonies were cultivated in 10 ml LB containing 100 μg/ml carbenicillin at 37 °C, 150 rpm until the OD reached 0.5 to 0.8 and the culture was induced with 0.6% w/v L-arabinose and grown overnight at 25 °C. 10^8^ cells from each induced single colony culture were pelleted and washed with either TBSC (calcium-containing buffer) or MBSE (chelator-containing buffer) before being resuspended in the same buffer but now also supplemented with either 150 nM of biotinylated mIgG1 or 500 nM of biotinylated hFab and incubated on a rotamixer at room temperature (RT) for 1 h. Following target incubation in the presence or absence of calcium, the cells were washed with TBSC/MBSE prior to being resuspended in 150 nM human serum albumin (HSA)-Alexa 647 conjugate (in-house produced) and 2 μg/ml streptavidin conjugated with R-phycoerythrin (SAPE, Invitrogen) and incubated dark on ice for 30 min. The cells were subsequently washed and lastly resuspended in cold TBSC/MBSE before being analyzed using a Gallios flow cytometer (Beckman Coulter).

### Subcloning, protein production, and purification

To produce and characterize promising candidates, single clone genes were amplified from the display vector into expression vector pET-45b(+) (MilliporeSigma) using primers to introduce an N-terminal His_6_-tag and the *XhoI* and *NcoI* restriction sites. The resulting plasmids were sequence-verified through Sanger sequencing by Eurofins (Luxembourg, Luxembourg). *E. coli* BL21(DE3) was used for production and the cells were subjected to sonication preparatory to purify the supernatant using table-top columns packed with IgG Sepharose 6 Fast Flow resin (Cytiva). The buffers used for purification were MBSC (25 mM MES, 150 mM NaCl, 1 mM CaCl_2_, pH 6) for equilibration, 5 mM NH_4_Ac supplemented with 1 mM CaCl_2_, pH 6 for washing, and 0.5 M HAc, pH 2.8 for elution. Upon elution, the samples were buffer exchanged on PD-10 Desalting columns (Cytiva) to TBSC and the final purity was analysed using SDS-PAGE.

### Circular dichroism (CD) spectroscopy

Analysis of secondary structure and stability was obtained through a circular dichroism spectrum ranging 195 nm to 260 nm measured at a protein concentration of 0.4 mg/ml at 20 °C followed by a variable temperature measurement (VTM) using a Chirascan instrument (Applied Photophysics Ltd). During the VTM, the ellipticity was measured at 221 nm while heating the sample from 4 to 95 °C using a temperature gradient of 5°C/min. The analysis was performed both in TBSC and TBS supplemented with 5 mM EDTA, pH 7. Fits of the VTM data to a two-state folding/unfolding model, as described in (29), provided the enthalpy of unfolding, the melting temperature (*T*_m_), and the fraction unfolded protein as a function of temperature.

### Size exclusion chromatography

Using a Bio-Rad NGC chromatography system (Bio-Rad) along with a Superdex 75 Increase 5/150 Gl column (Cytiva) it was investigated whether the variants were prone to aggregate similarly as to in a previous study (18).

### Surface plasmon resonance (SPR)

The kinetic values of the native targets mIgG1, human polyclonal Fab-, and human polyclonal Fc were determined using a Biacore T200 instrument (Cytiva). By amine coupling, each target was diluted in 10 mM NaAc, pH 4.5, and immobilized in separate flow cells on a Series S CM5-chip. Single-cycle kinetics was obtained at 25 °C through injection of a two-fold dilution series (2000 nM, 1000 nM, 500 nM, 250 nM) at a flow rate of 30 μl/min. The experiment was performed both with TBSCT (TBSC, 0.05% (v/v) Tween20, pH 7) and TBSET (TBS, 100 mM EDTA, 0.05% (v/v) Tween20, pH 7) as running buffer and after each cycle the surfaces were regenerated with 10 mM HCl. The responses obtained were double-referenced against a blank flow cell and a corresponding buffer injection preceding the determination of affinity constants applying a 1:1 Langmuir binding model.

### Nuclear magnetic resonance

Double ^15^N/^13^C labeled proteins were produced and purified as previously described except for being cultivated in M9 minimal media according to published protocols (^15^NH_4_Cl: 1 g/L and ^13^C d-glucose: 4 g/L) (30, 31).

NMR experiments were recorded on a Bruker Avance 850-MHz and 600-MHz NMR spectrometer equipped with triple-resonance cryogenic probes (Bruker, Billerica, MA, US). For backbone assignments (^15^N, ^13^Cα, ^13^C_β_, ^13^C’, ^1^H_α_, ^1^H_β_, and ^1^H_N_), a series of triple-resonance experiments, namely, HNCA, HN(CO)CA, HNCACB or CBCANH, CBCA(CO)NH, HNCO, and HN(CA)CO were performed at 293K using uniformly [^13^C/^15^N]-labeled C2_wt_, C2_Ca V3_ (−) Ca^2+^ and C2_Ca V3_ (+) Ca^2+^ proteins. 3D ^15^N-Edited TOCSY and NOESY experiments with 80 ms spin-lock and 150 ms mixing times were also acquired to assist in the assignment. NMR spectra were processed using NMRPipe and NMRDraw software and visualized and analyzed using ANSIG for Windows (32) and CCPN 2.1.5. Sequential backbone-NMR assignments were performed manually using the interresidual through-bond connectivities from triple resonance experiments (BMRB access code 52448. Assigned chemical shifts were directly referenced against 4, 4-dimethyl-4-silapentane-1-sulfonic acid for the ^1^H atoms, whereas ^13^C and ^15^N atoms were referenced indirectly (http://www.bmrb.wisc.edu). All relaxation experiments were recorded on the Bruker Avance 850-MHz NMR spectrometer. Protein concentrations of 1.68 mM, 0.7 mM and 0.4 mM were used for C2_wt_, C2_Ca V3_ (−) Ca^2+^ and C2_Ca V3_ (+) Ca^2+^ respectively, for all NMR experiments. 30 mM MES NMR buffer at pH 6.0 with D_2_O [10% (v/v)] was added to all NMR samples for the field-frequency lock. Relaxation spectra were processed using NMRPipe and NMRDraw software (33) and visualized and analyzed using Sparky (v3.113; https://www.cgl.ucsf.edu/home/sparky; UCSF).

The 2D HSQC-based ^15^N-relaxation experiments, namely T_1_, T_2_, and ^15^N-{^1^H}-heteronuclear nuclear Overhauser effects (hetNOEs) were collected in an interleaved manner. Delays of 50, 100, 200, 500, 800, 1000, 1200 and 1500 ms were used for T_1_ measurements, and 16.96, 33.92, 50.88, 84.80, 101.76, 118.72, 135.68, 152.64, 169.60 (duplicate), 186.56, 203.52, 220.48, 254.4 and 288.32 ms were used for T_2_ measurements (34) Relaxation delays of 3 s and 2 s were set for T_1_ and T_2_ experiments, respectively. Steady-state ^15^N-{^1^H}-heteronuclear NOE spectra were measured with either 5-s delays between each free-induction decay or 2-s delays, followed by a 3-s series of 120° nonselective ^1^H pulses as previously described (35). All the relaxation experiments were performed with time-domain sizes of 256(^15^N) × 2048(^1^H) complex points and sweep widths of 11,029.412 Hz and 2929.115 Hz for C2_Ca V3_ (−) Ca^2+^ and C2_Ca V3_ (+) Ca^2+^, and 11,029.412 Hz and 2411.963 Hz for C2_wt_, along the ^1^H and ^15^N dimensions respectively, with 8 scans for T_1_ or T_2_ and 24 scans for the ^15^N-{^1^H}-heteronuclear NOE measurements.

#### ^15^N-relaxation data analysis

SPARKY (v3.113; https://www.cgl.ucsf.edu/home/sparky; UCSF, San Francisco, CA, USA) was used to analyze the ^15^N-relaxation data of C2_wt_, C2_Ca V3_ (−) Ca^2+^ and C2_Ca V3_ (+) Ca^2+^. Peak heights of the ^1^H-^15^N cross-peaks in the T_1_ and T_2_ spectra were measured using a peak-picking routine in SPARKY and fitted to a single exponential-decay function using the Curvefit module in SPARKY:

(1) $$I\left(t\right)=I_{0}\times e^{\left(-\frac{t}{Td}\right)}$$

Where I(t) represents the intensity of the signal at time t, *I*_*0*_ represents the intensity at time t = 0, and *T*_*d*_ represents the decay constant for T_1_ or T_2_. Errors in T_1_ and T_2_ were estimated from the fittings using 500 Monte Carlo simulations. ^15^N-{^1^H}-heteronuclear NOE values were calculated from the ratio of peak intensities, *I*_*sat*_*/I*_*unsat*_, where *I*_*sat*_ and *I*_*unsat*_ represent the intensities of peaks in saturated and unsaturated spectra, respectively. The NOE error (σ_NOE_) was calculated as:

(2) $$\sigma_{NOE}=\frac{I_{sat}}{I_{unsat}}\;{\left[{\left(\frac{\sigma_{sat}}{I_{sat}}\right)}^{2}+{\left(\frac{\sigma_{unsat}}{I_{unsat}}\right)}^{2}\right]}^{1/2}$$

Where σ_sat_ and σ_unsat_ represent the root mean square variation in the noise in empty spectral regions of the spectra in the presence and absence of proton saturation, respectively.

#### Model-free analysis

Internal motion parameters were determined according to model-free formalism (36) using Modelfree4 software (v4.20; Columbia University, New York, NY, USA). T_1_, T_2_, and heteronuclear NOE-relaxation data were optimized with an isotropic diffusion model using 500 Monte Carlo simulations and assuming an internuclear distance (r_NH_) of 1.02 Å and chemical-shift anisotropy of −160 ppm for the ^15^N nucleus. The overall motion of the protein in the isotropic diffusion model is characterized by the overall correlation time, τ_m_, and its spectral-density function is represented as:

(3) J(ω)=S2τm(1+ω2τm2)+(1−S2)τ(1+ω2τ2)

with

$$\frac{1}{\tau }=\frac{1}{\tau_{m}}+\frac{1}{\tau_{e}}$$

where τ_e_ represents the correlation time for internal motions, and $S^{2}=S_{f}^{2}\;S_{S}^{2}$ represents the generalized order parameter. $S_{f}^{2}$ is the generalized order parameter for fast internal motion on the sub-ns time scale, and $S_{S}^{2}$ is the order parameter for slow internal motion on the ns time scale (19).

After deriving a global τ_m_, S^2^ and τ_e_ was fitted against the experimental data until an acceptable solution was obtained by minimization of the target function, χ^2^:

(4) $$\chi^{2}=\sum \left[\;\left(T_{1}^{\exp\;}-T_{1}^{cal}\right)/\sigma_{1}^{2}+\left(T_{2}^{\exp\;}-T_{2}^{cal}\right)/\sigma_{2}^{2}+\left({NOE}_{2}^{\exp\;}-{NOE}_{2}^{cal}\right)/\sigma_{N}^{2}\right]$$

In this equation, the summation extends over all the amino acid residues, with the superscripts indicating either experimental or calculated values and sigma representing the standard error of the parameter.

### CS_Rosetta modelling of C2_Ca V3_ (+) Ca^2+^

The structure of C2_Ca V3_ was calculated with chemical shift Rosetta (22). The fragment generation was based on the following nuclei: H^α^, H^N^, C^α^, C^β^ and C′ and a pool of 3000 conformers were obtained. The calculations have converged based on the analysis of Cα-RMSD of each structure to the lowest-energy structure. The final NMR ensemble with 10 models of the lowest energy was validated and deposited in the PDB database (access code 9FA8).

### Molecular dynamics (MD) simulations

The structure determined with CS-Rosetta was used to set up the simulation system. Ionizable residues, including histidine, were protonated based on their pKa values in water. We positioned a calcium ion within 5 Å of four aspartate residues (Asp 23, 27, 31, and 34) potentially involved in coordination with the calcium ion. Additional Na^+^ and Cl^-^ ions were placed at random positions to neutralize the system to achieve a concentration of 150 mM NaCl. The system was then solvated with a 72 Å cubic box of TIP3P water (37), resulting in a total of 34,688 atoms. An initial energy minimization of 6000 steps was performed, followed by heating to 300.0 K for 24 ps and equilibration at 300.0 K for 76 ps. Throughout the heating and equilibration, we imposed harmonic restraints on the protein backbone atoms and distance restraints between the four Asp residues and the calcium ion. The distance restraints, in particular, were only applied when the distance to the calcium ion exceeded 5 Å. Following equilibration, a 1 μs MD simulation was performed at 300.0 K and 1 bar without positional restraints on the protein backbone atoms, while maintaining the distance restraints (to the calcium ion). The CHARMM 36m force fields (38) were used to describe the protein and the calcium, Na^+^ and Cl^-^ ions and the TIP3P water potential was used to represent the water molecules. For the electrostatic interactions, the particle mesh Ewald (PME) summation method (39) was used with a 12 Å cut-off for the real-space interactions and the van der Waals interactions. The MD simulation was performed using the leap-frog Verlet integrator with 2 fs time integration and SHAKE (40) applied to constrain bonds with any hydrogen atom. The temperature and pressure of the system were controlled using the Nose-Hoover (41) and Andersen’s extended system algorithms (42). All simulations were performed with the CHARMM program (43) using the DOMDEC-GPU module for GPU acceleration (44).

## Data deposition

The atomic coordinates for the CS-Rosetta-derived structure of C2_Ca V3_ in the presence of calcium are deposited in the Worldwide Protein Data Bank *via* the Protein Data Bank in Europe (PDBe) with accession ID: 9FA8. Resonance assignments of C2_Ca V3_ in the presence of calcium are deposited in the Biological Magnetic Resonance Data Bank (BMRB) under entry “52448”.

## Data availability

All other data are contained within the manuscript.

## Supporting information

This article contains supporting information.

## Conflict of interest

S. H. and M. J. have filed a patent application regarding use of the domain as a purification tool.
