# Supplementary material for: Cooperative folding as a molecular switch in an evolved antibody binder
Source: J Biol Chem. 2024 Sep 19;300(11):107795. doi: 10.1016/j.jbc.2024.107795 (PMC11532951; doi:10.1016/j.jbc.2024.107795)
Supplement: Supporting Figures and Tables [file mmc1.docx]

**Supplementary material**

**Cooperative Folding as a Molecular Switch in an Evolved Antibody Binder**

Malin Jönsson^1^, Ameeq Ul Mushtaq^2^, Tamás Milán Nagy^2^, Emma von Witting^1^, John Löfblom^1^, Kwangho Nam^3^, Magnus Wolf-Watz^2♯^, Sophia Hober^1♯^

^1^Department of Protein Science, KTH-Royal Institute of Technology, SE-10691, Stockholm, Sweden

^2^Department of Chemistry, Umeå University, SE-901 87, Umeå, Sweden

^3^Department of Chemistry and Biochemistry, University of Texas at Arlington, Arlington, Texas 76019, USA

^♯^Corresponding authors: sophia@kth.se, phone +46 8 790 87 94, fax +46-5537-8481,

magnus.wolf-watz@umu.se, phone +46 90 786 76 90

**
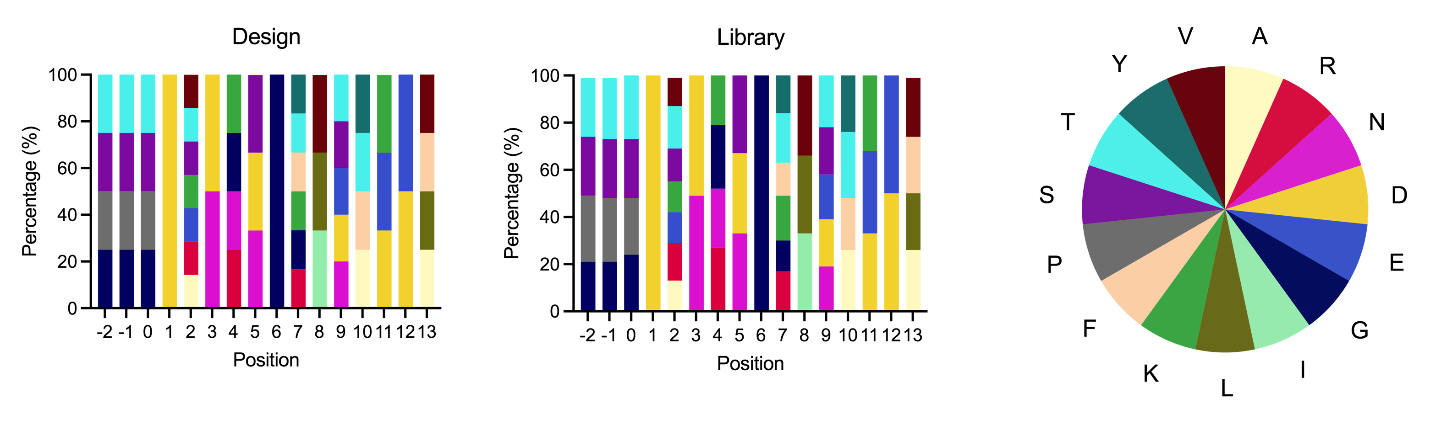
**

**Figure S1.** **Deep sequencing confirms that the experimental library is as designed.** Next generation sequencing data showing a high similarity in the amino acid distribution of the designed (left graph) and the experimental library (right graph) with the corresponding allowed amino acids illustrated in a pie chart.


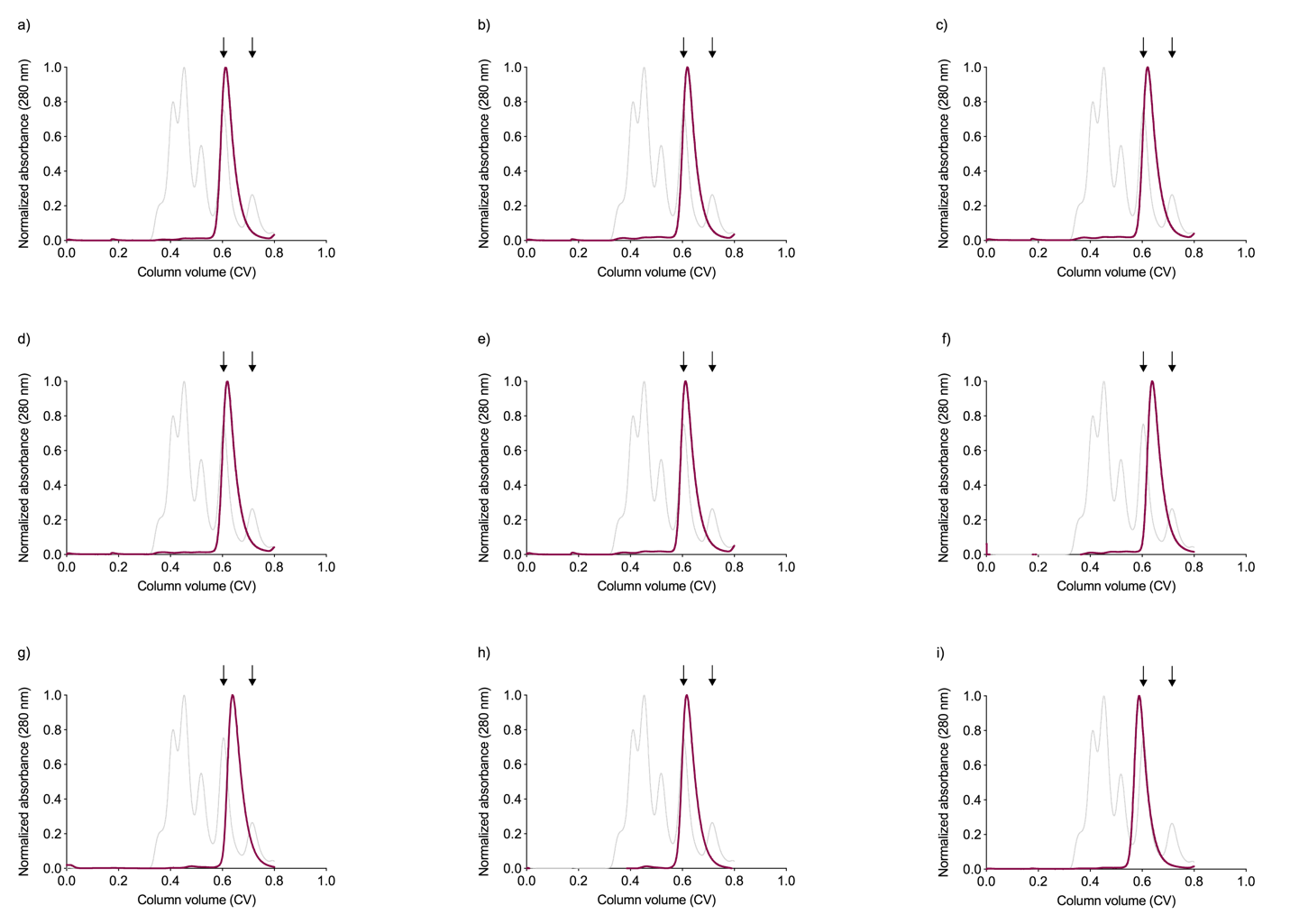


**Figure S2.** **SEC chromatograms for all characterized variants (burgundy) confirming expected molecular weight (approximately 8.5 kDa) and monomeric form.** Indicated by arrows is the molecular size of two reference proteins (13.7 kDa and 6.50 kDa) within the calibrant (grey) for comparison. a) C2_Ca V1_, b) C2_Ca V2_, c) C2_Ca V3_, d) C2_Ca V4_, e) C2_Ca V5_, f) C2_Ca V6_, g) C2_Ca V7_, h) C2_Ca V8_, i) C2_wt_.

**Table S1. SPR-measured kinetics of all characterized variants and the wild type.** The evolved variants are showing similar kinetic values in the presence of calcium as the parental domain (C2_wt_) while no detectable signals could be measured for the evolved variants in the absence of calcium (see Figure 3, Figure S3-S7). The rate of complex formation is represented as the association constant k_a_ and the rate of complex decay is represented by the dissociation constant k_d_. The affinity constant is calculated from the ratio of k_d_/k_a_. R_max_ is the maximum analyte binding capacity of the surface and Chi^2^ is showing the confidence of the fit by averaging the difference between the experimental data and the fitted 1:1 binding model, thus it should be as low as possible for higher confidence in the calculated kinetic parameters. It should be noted that the k_d_ value is below the instrument’s lower limit of detection (0.5 s^-1^) for most variants (marked with *) which results in a large variation in calculated K_D_. However, it is clear that a target association (k_a_) can be measured for the variants in the presence of calcium while this is not possible, except for the wildtype C2, in the absence of calcium (Figure 3, Figure S3-S7).

**
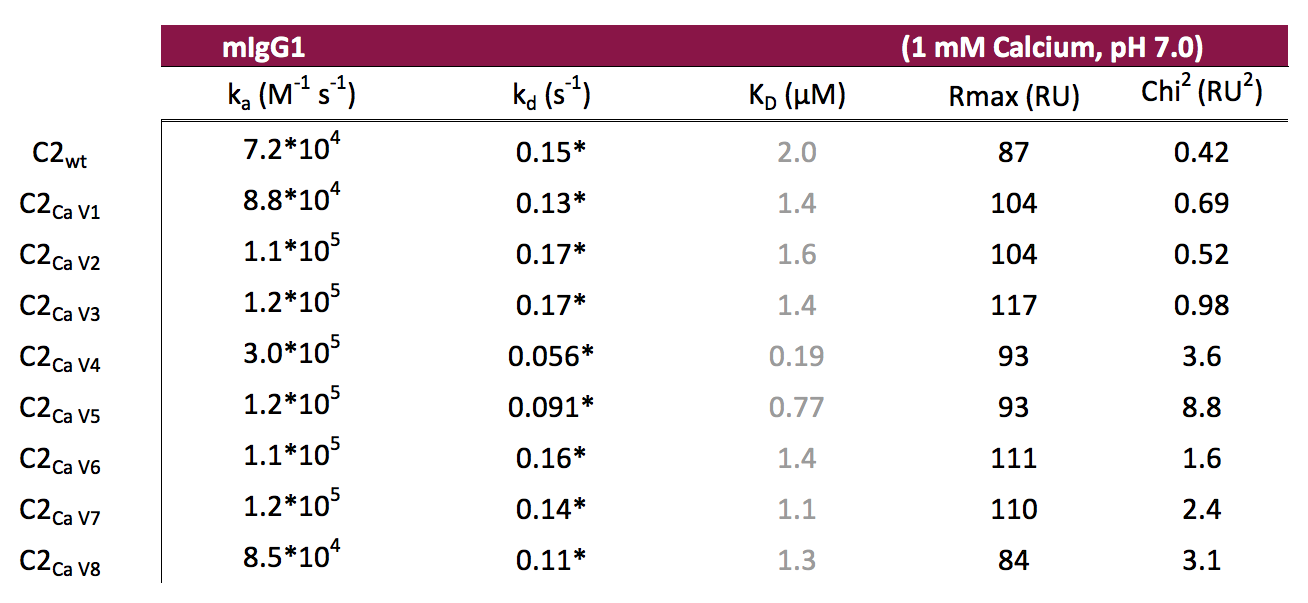
**

**
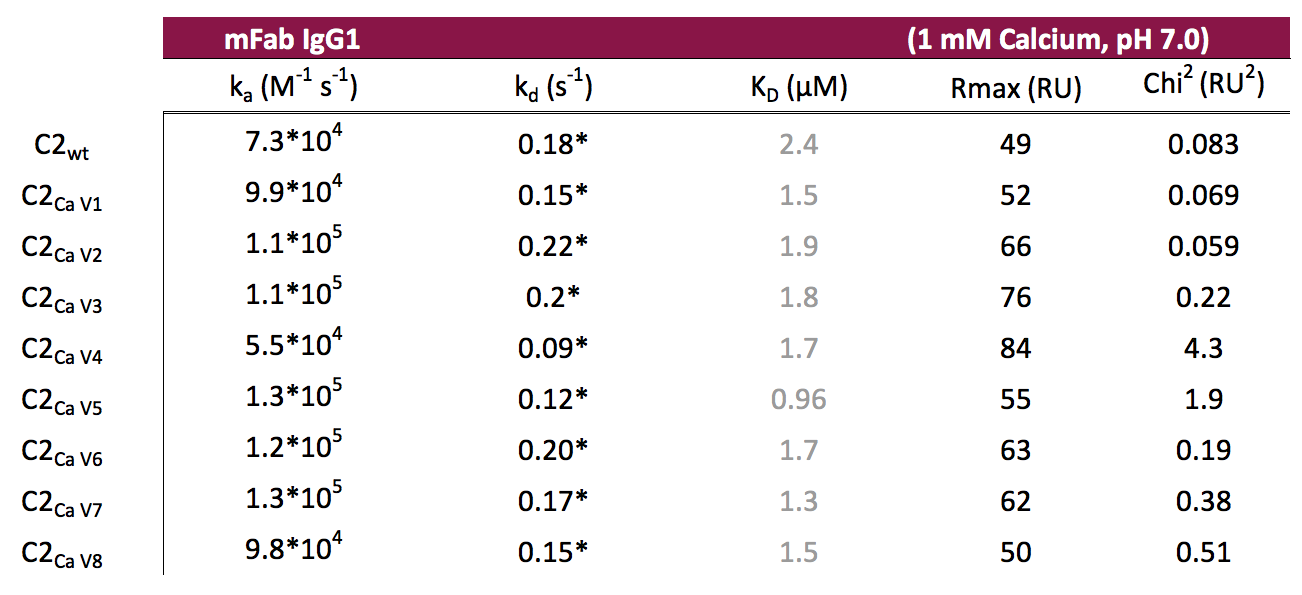
**

**
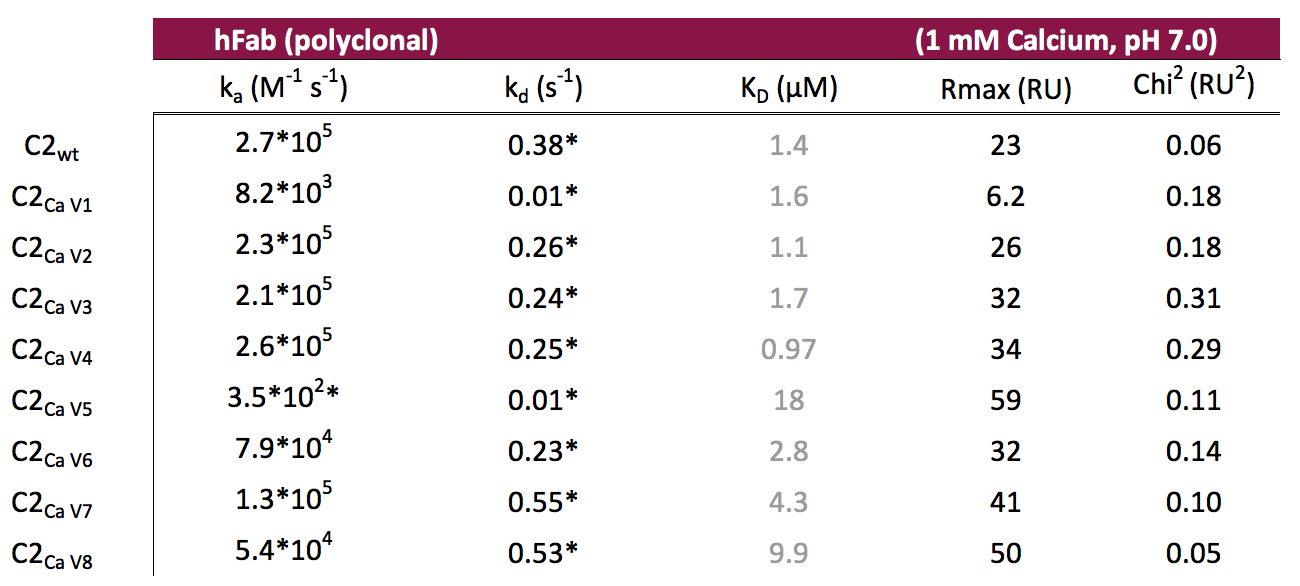
**

**
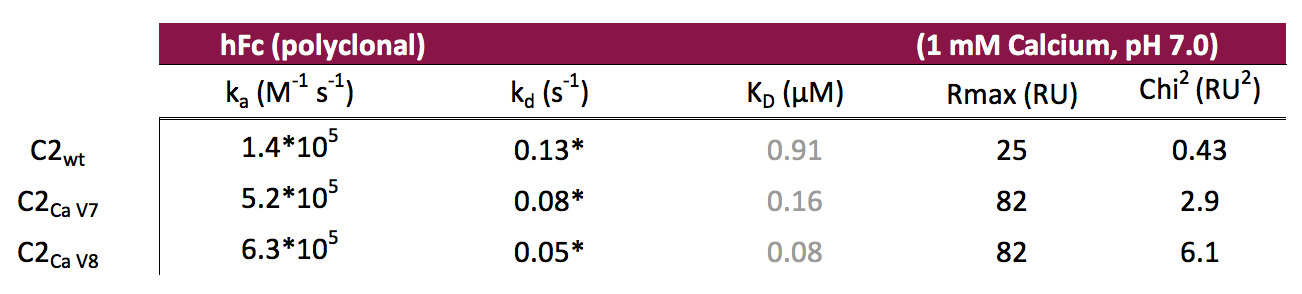
**

**
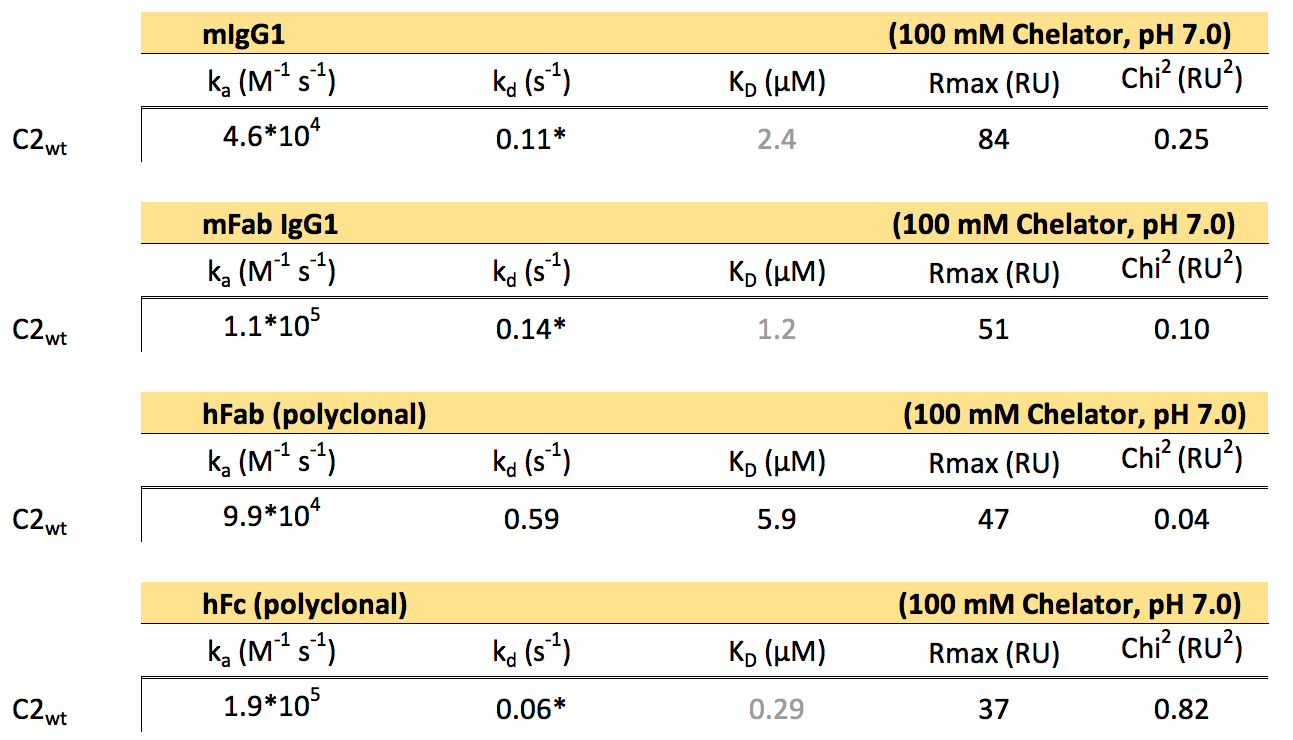
**

**
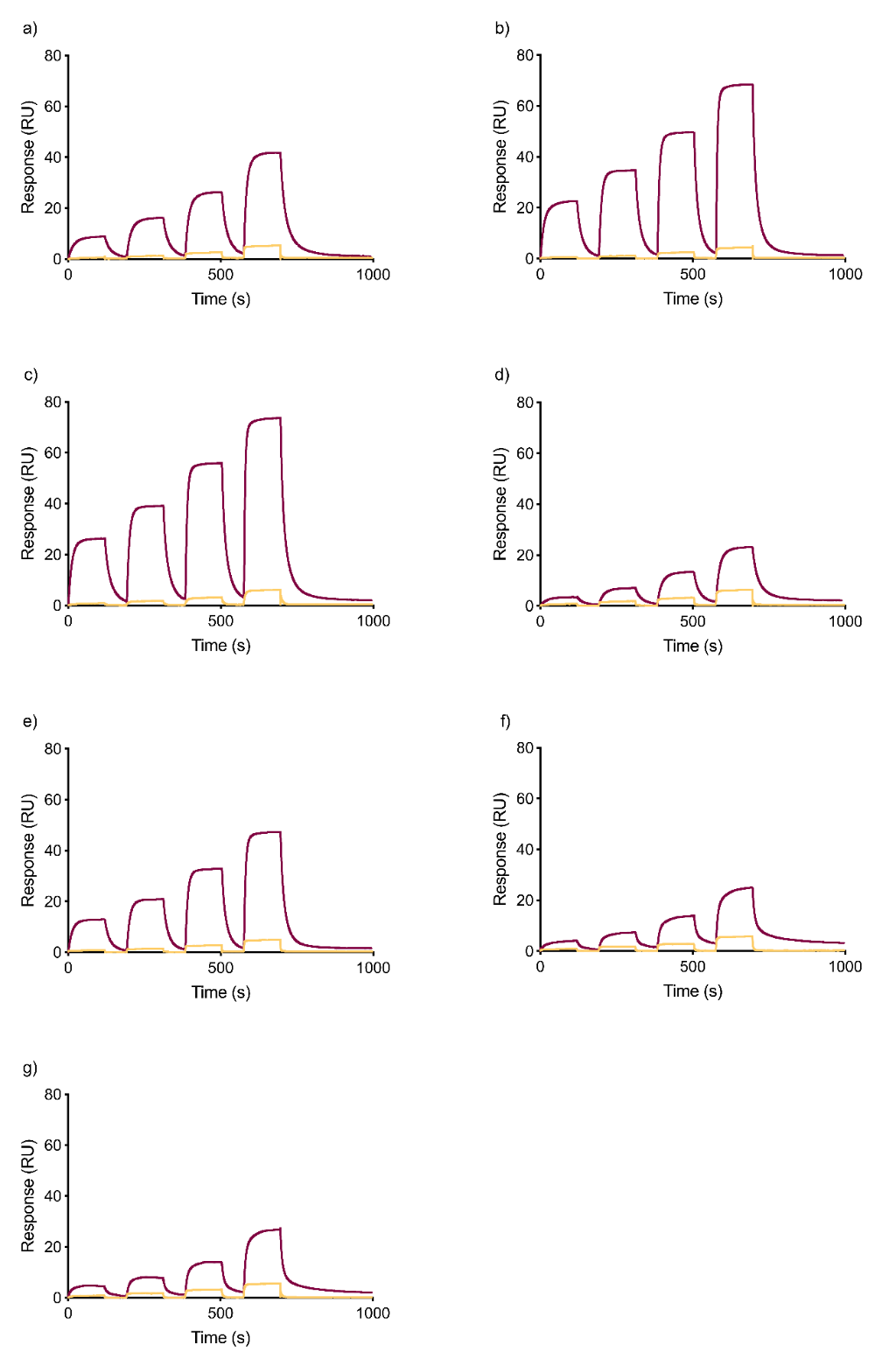
**

**Figure S3.** **SPR-measured target (mouse IgG1) interaction of the characterized variants in the presence (burgundy) or absence (yellow) of calcium.** During the injected concentration series of 250 nM, 500 nM, 1000 nM, and 2000 nM, all variants display calcium-dependency seemingly to a varying degree although it was not possible to determine a kinetic association constant (k_a_) in the absence of calcium for any of the variants. a) C2_Ca V1_, b) C2_Ca V2_, c) C2_Ca V4_, d) C2_Ca V5_, e) C2_Ca V6_, f) C2_Ca V7_, g) C2_Ca V8_.


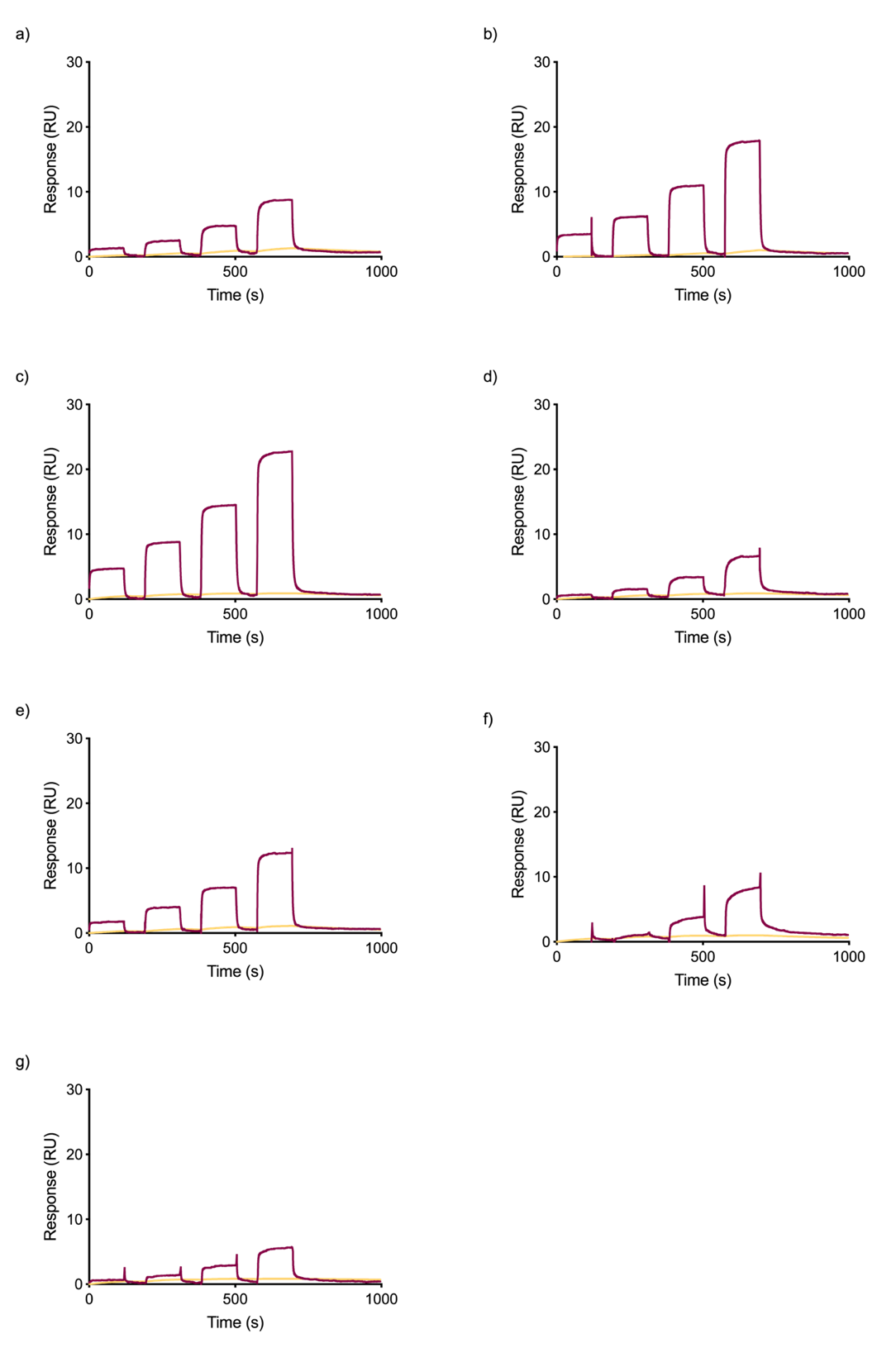


**Figure S4.** **SPR-measured target (polyclonal human Fab) interaction of the characterized variants in the presence (burgundy) or absence (yellow) of calcium.** During the injected concentration series of 250 nM, 500 nM, 1000 nM, and 2000 nM, all variants display calcium-dependency and it was not possible to determine a target association constant (k_a_) in the absence of calcium for any of the variants. a) C2_Ca V1_, b) C2_Ca V2_, c) C2_Ca V4_, d) C2_Ca V5_, e) C2_Ca V6_, f) C2_Ca V7_, g) C2_Ca V8_.


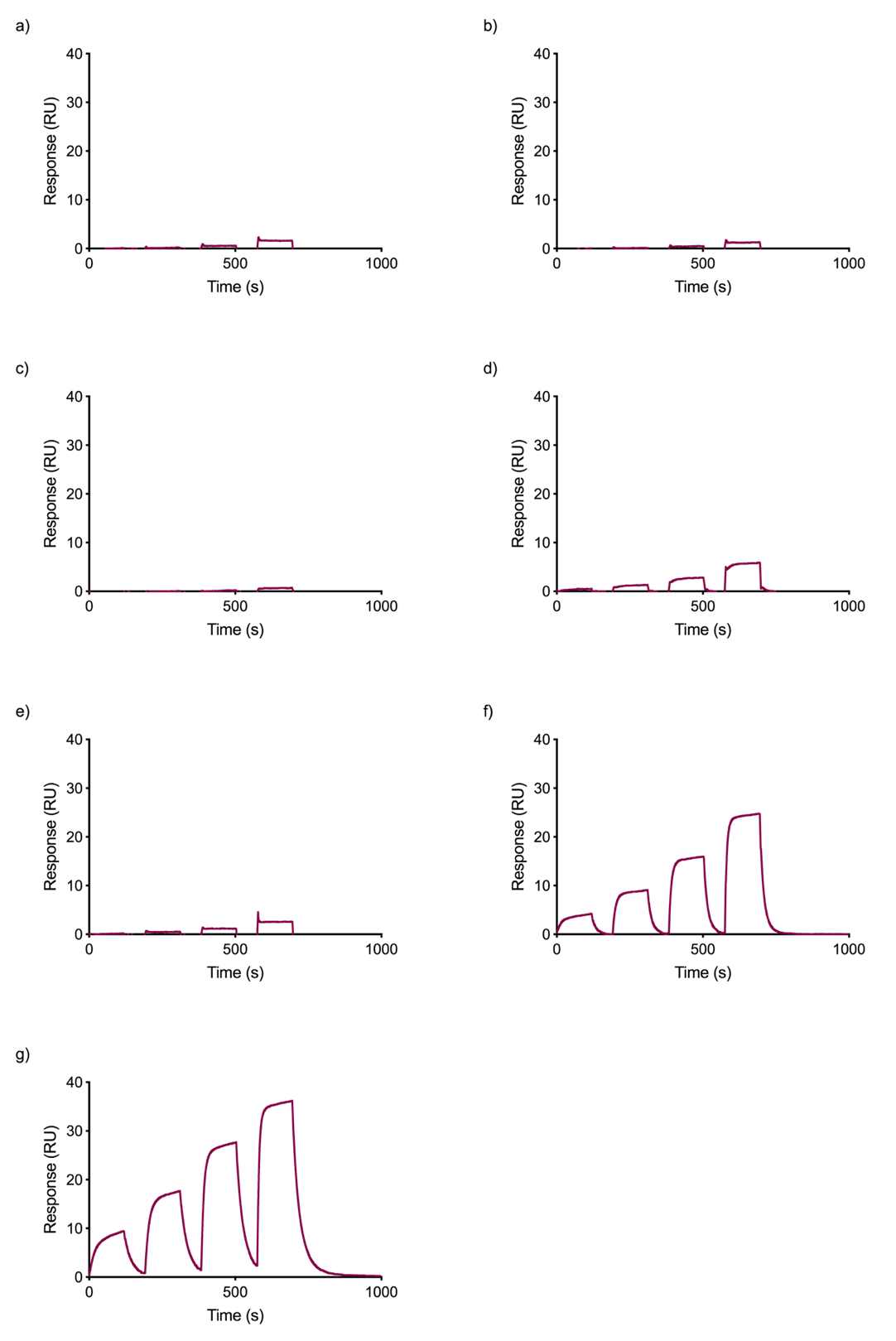


**Figure S5.** **SPR-measured target (human Fc) interaction of the characterized variants in the presence (burgundy) of calcium.** During the injected concentration series of 250 nM, 500 nM, 1000 nM, and 2000 nM, only C2_Ca V7_ and C2_Ca V8_ display a retained Fc-interaction where a target association constant (k_a_) could be measured. a) C2_Ca V1_, b) C2_Ca V2_, c) C2_Ca V4_, d) C2_Ca V5_, e) C2_Ca V6_, f) C2_Ca V7_, g) C2_Ca V8_.


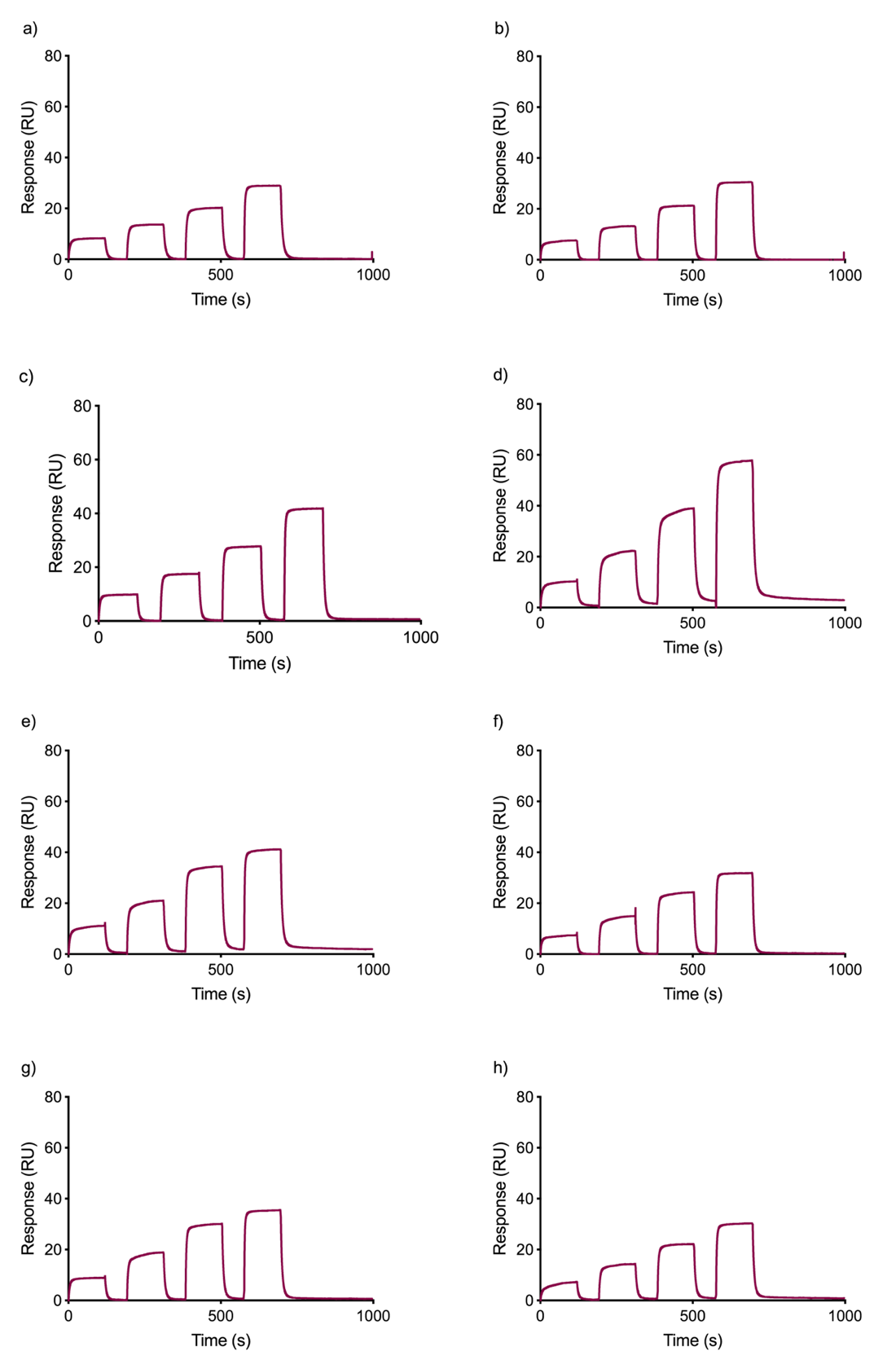


**Figure S6.** **SPR-measured target (mouse Fab IgG1) interaction of the characterized variants in the presence (burgundy) of calcium.** During the injected concentration series of 250 nM, 500 nM, 1000 nM, and 2000 nM, all variants display retained Fab-binding with measurable association constants (k_a_). a) C2_Ca V1_, b) C2_Ca V2_, c) C2_Ca V3_, d) C2_Ca V4_, e) C2_Ca V5_, f) C2_Ca V6_, g) C2_Ca V7_, h) C2_Ca V8_.


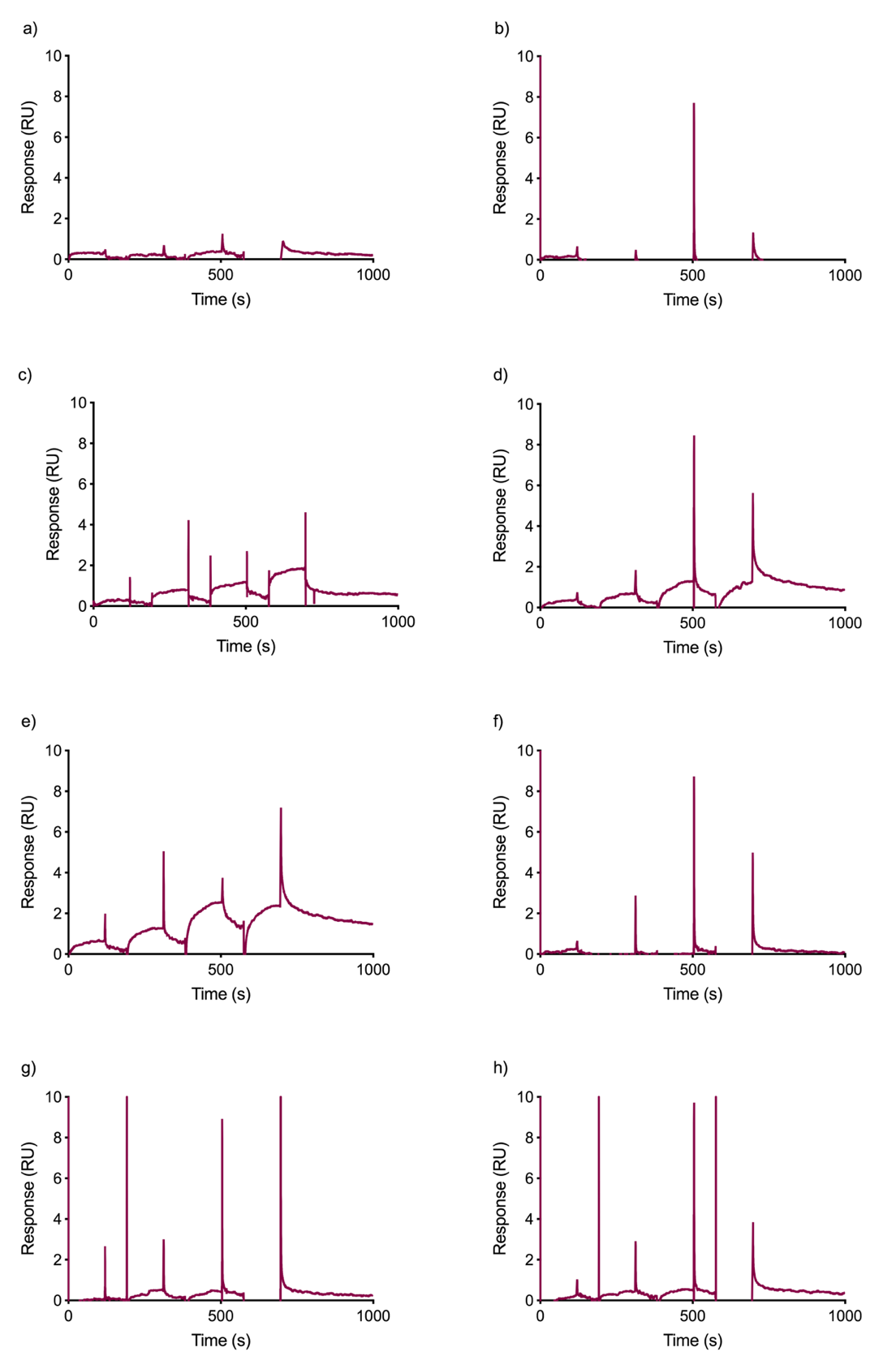


**Figure S7.** **SPR-measured target (mouse Fc IgG1) interaction of the characterized variants in the presence (burgundy) of calcium.** During the injected concentration series of 250 nM, 500 nM, 1000 nM, and 2000 nM, all variants display a lost affinity for Fc. a) C2_Ca V1_, b) C2_Ca V2_, c) C2_Ca V3_, d) C2_Ca V4_, e) C2_Ca V5_, f) C2_Ca V6_, g) C2_Ca V7_, h) C2_Ca V8_.


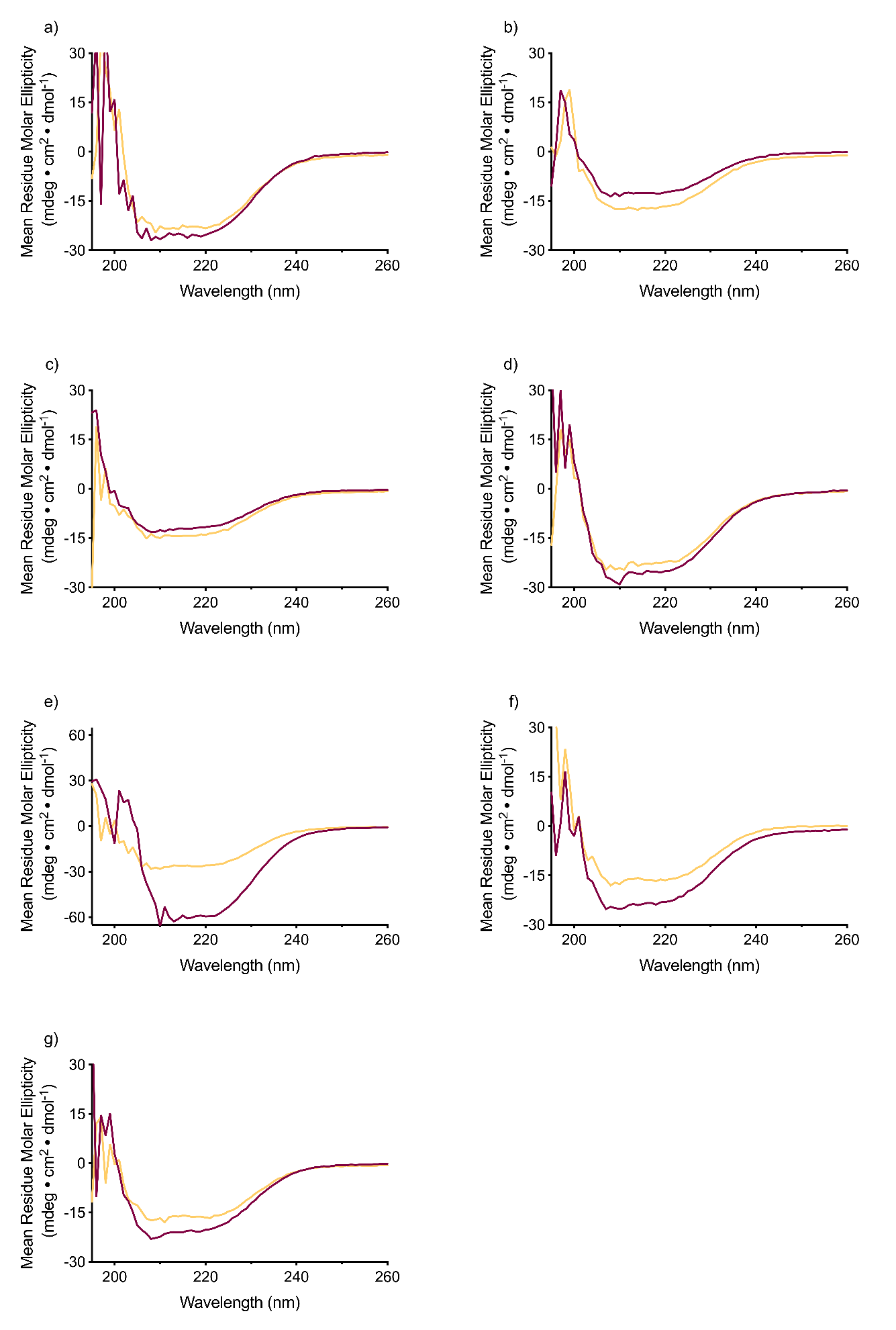


**Figure S8.** **Circular dichroism spectra of all characterized variants in either calcium buffer (burgundy) or chelating buffer (yellow).** Most of the variants display a tendency to be less structured in the absence of calcium. a) C2_Ca V1_, b) C2_Ca V2_, c) C2_Ca V4_, d) C2_Ca V5_, e) C2_Ca V6_, f) C2_Ca V7_, g) C2_Ca V8_.

**
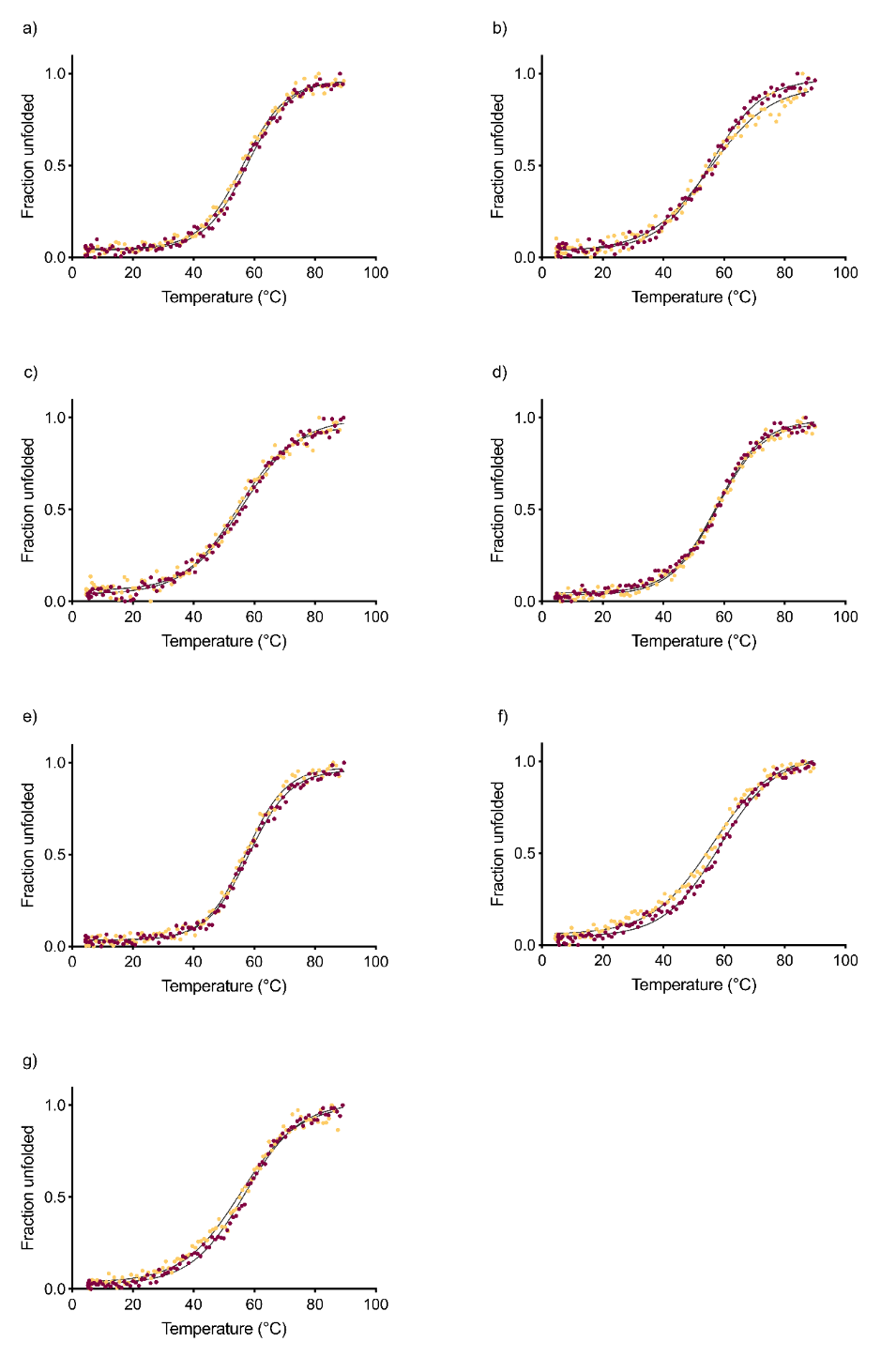
**

**Figure S9.** **Variable temperature measurements between 4°C and 95°C of all characterized variants in the presence (burgundy) or absence (yellow) of calcium.** All variants show a decreased thermal stability compared to the wildtype with approximately 10 °C and some display a broader temperature interval for unfolding in the absence of calcium. a) C2_Ca V1_ – T_m, Ca_ = 58 °C and T_m, Chelator_ = 56 °C, b) C2_Ca V2_ – T_m, Ca_ = 55 °C and T_m, Chelator_ = 55 °C, c) C2_Ca V4_ – T_m, Ca_ = 56 °C and T_m, Chelator_ = 55 °C, d) C2_Ca V5_ – T_m, Ca_ = 58 °C and T_m, Chelator_ = 57 °C, e) C2_Ca V6_ – T_m, Ca_ = 58 °C and T_m, Chelator_ = 57 °C, f) C2_Ca V7_ – T_m, Ca_ = 58 °C and T_m, Chelator_ = 56 °C, g) C2_Ca V8_ – T_m, Ca_ = 57 °C and T_m, Chelator_ = 55 °C.

**
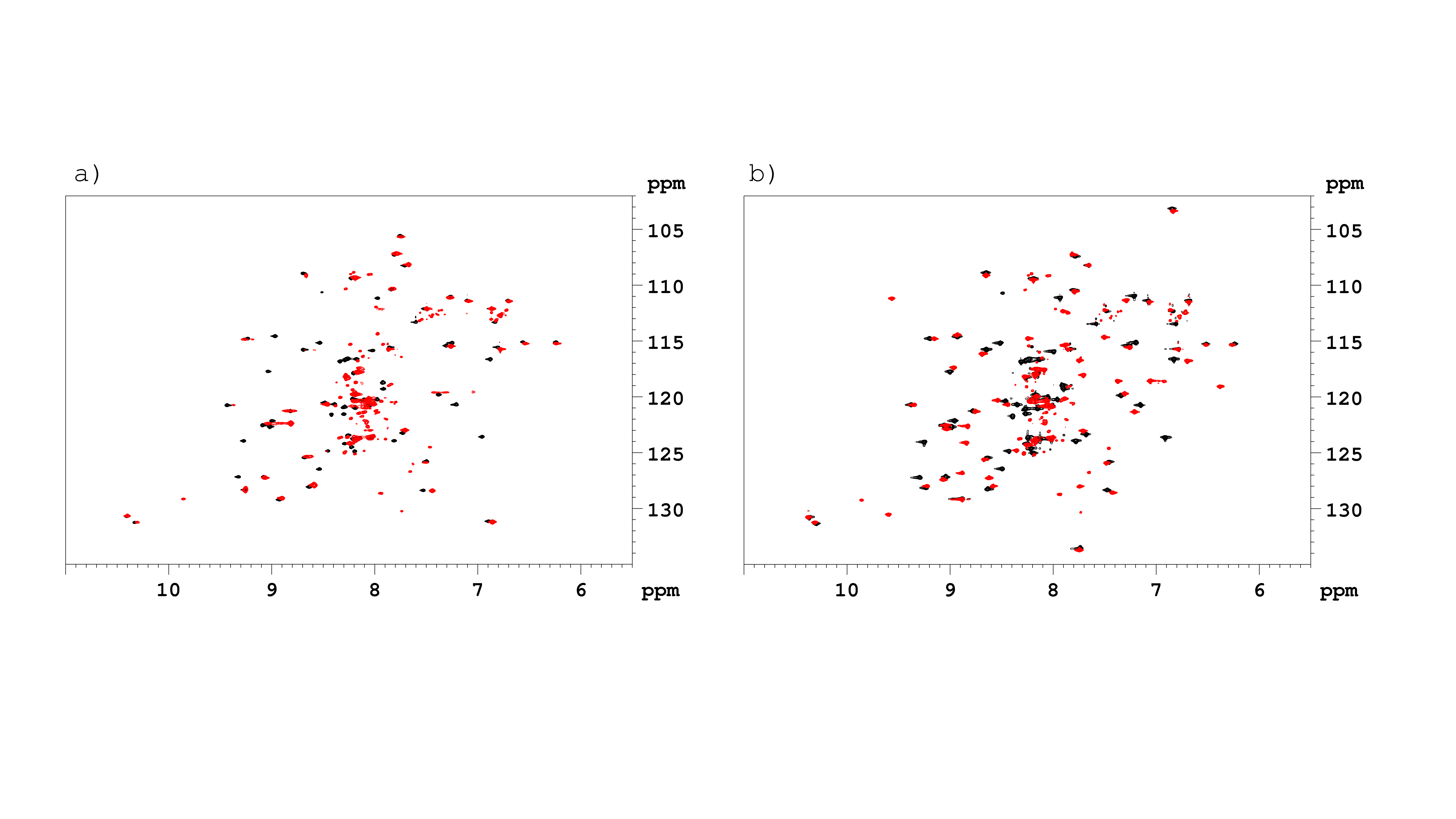
**

**Figure S10.** **C2_Ca V3_ is partially disordered in the absence of calcium.** Shown is a superimposition of ^1^H-^15^N HSQC spectra of the parental wild type (black contours) and C2_Ca V3_ (red contours) both with the absence of calcium (a) and with calcium (b). The clustering of resonances in the centrum of the spectra without calcium (a) indicates that C2_Ca V3_ is partially disordered. Upon adding calcium resonances spread out and become similarly dispersed to that of the wild type (b).


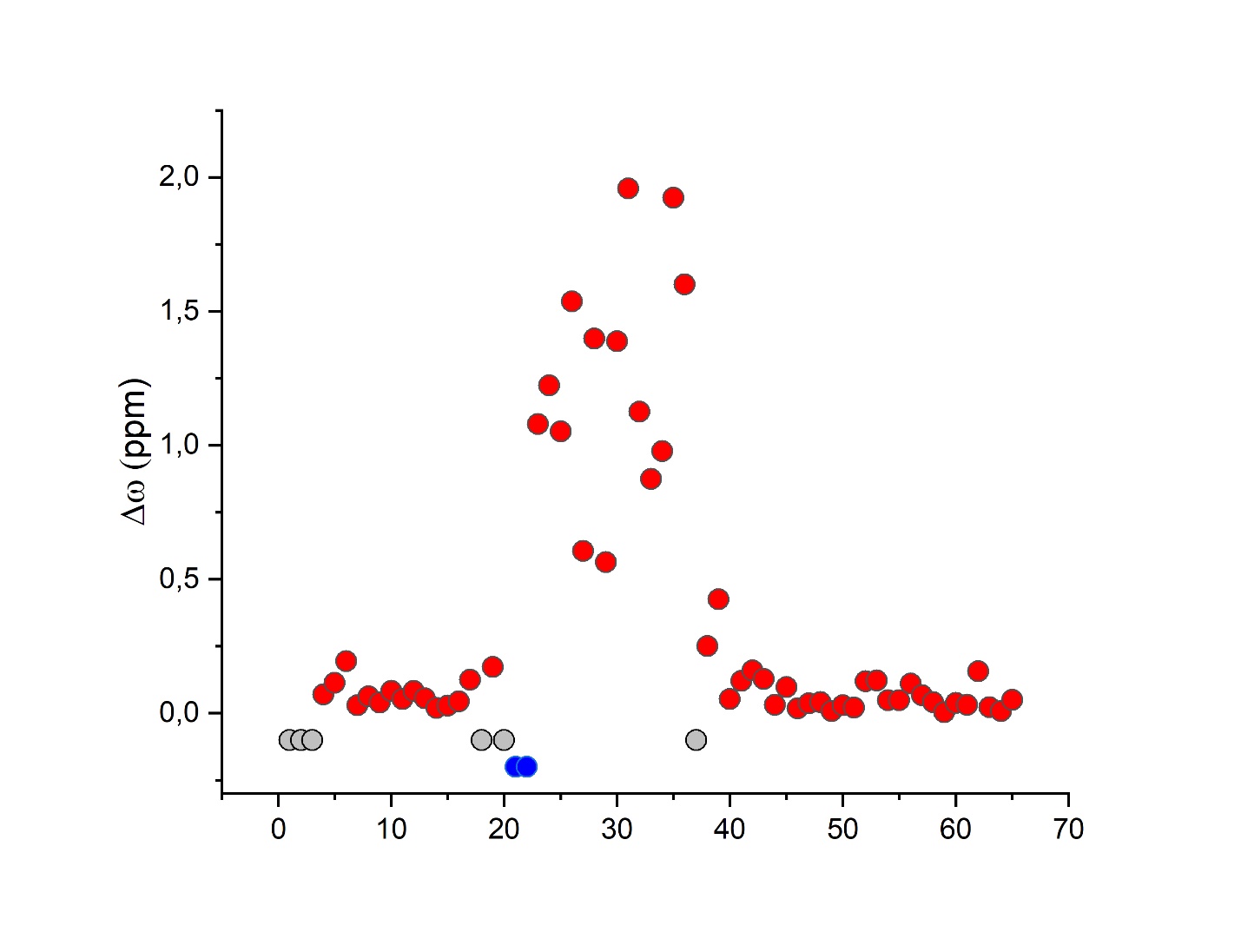


**Figure S11.** **Chemical shift difference upon calcium binding.** Shown is the weighted ^1^H and ^15^N chemical shift difference between calcium bound and apo C2_Ca V3_ displayed against primary sequence. The chemical shift difference is calculated according to: $\Delta\omega=\sqrt{(\frac{\Delta^{15}N}{5})^{2+(\Delta^{1}H)^{2}}}$ . Unassigned residues and proline residues are indicated with gray and blue circles, respectively.


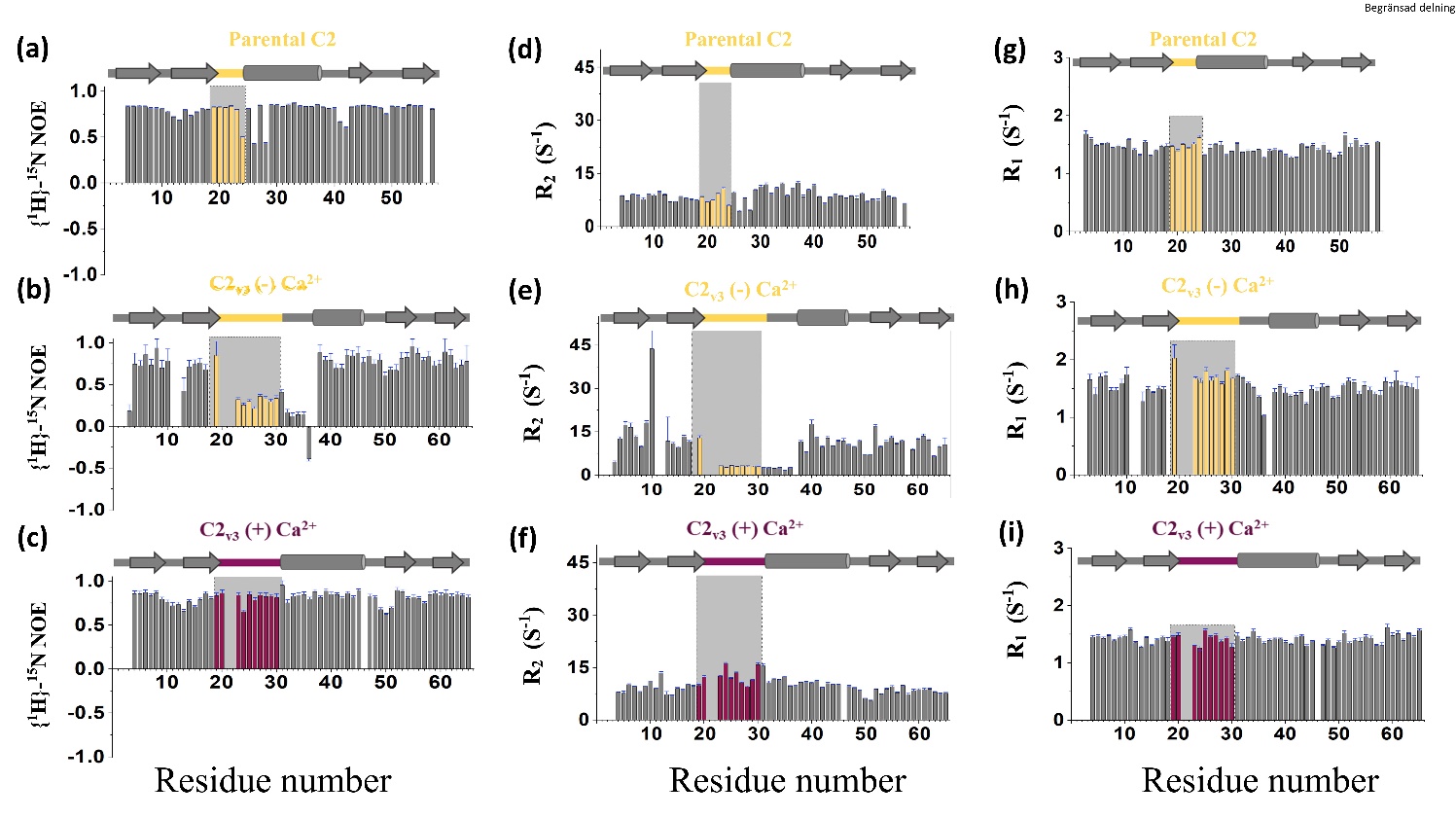


**Figure S12.** **NMR spin relaxation of C2 in absence and C2_Ca V3_ in absence and presence of calcium.** The spin relaxation rates used for computation of order parameters shown in Figure 5 are shown for the parental C2 domain (top row), C2_Ca V3_ in absence of calcium (middle row) and C2_Ca V3_ in presence of calcium (bottom row). Relaxation rates are shown for {^1^H}-^15^N heteronuclear NOEs (a-c), R_2_ (d-f) and R_1_ (g-i). The position of the loop in the parental C2 domain is coloured in yellow (top tow), and the position of the engineered loop in C2_Ca V3_ is shown in yellow middle row (absence of calcium) and burgundy bottom row (presence of calcium).


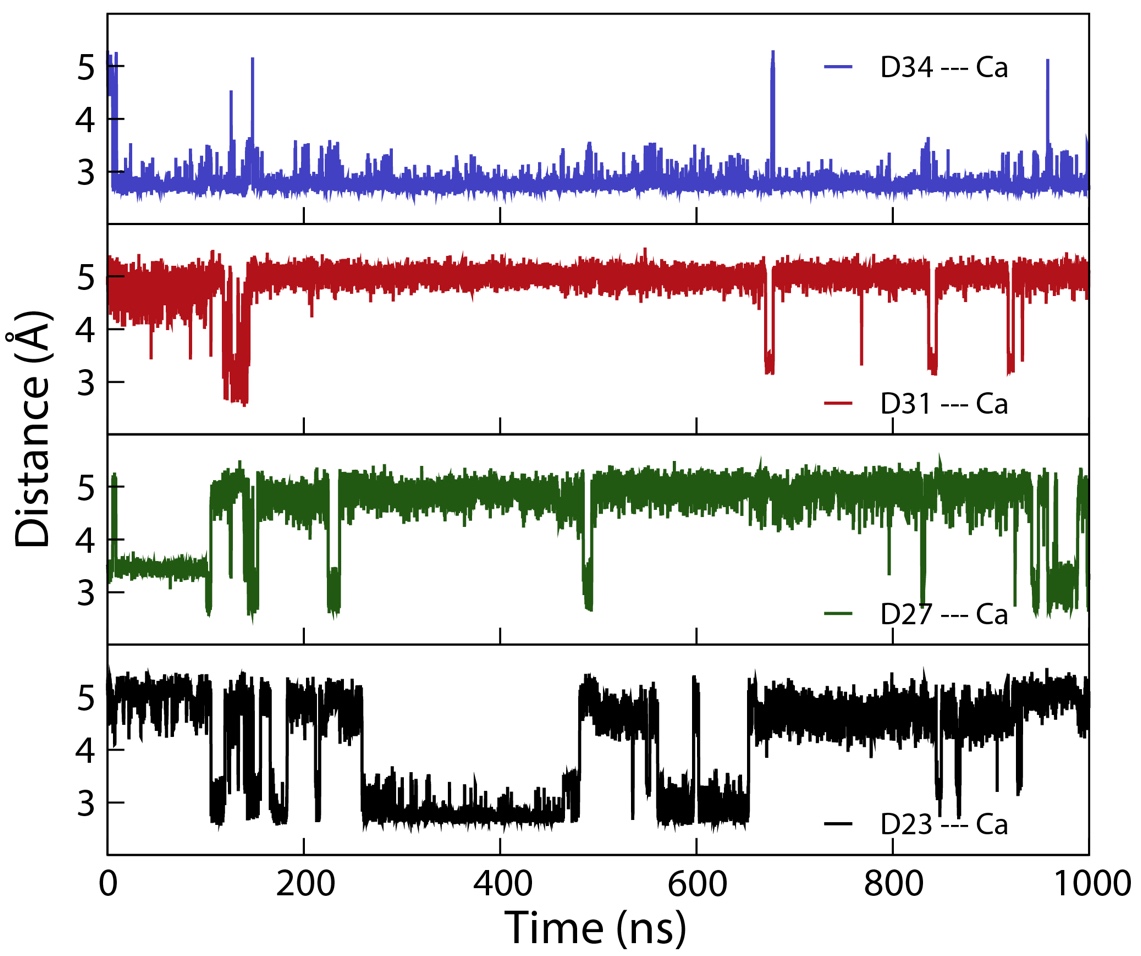


**Figure S13.** **Time evolution of Asp coordination to calcium.** Monitoring of the coordination distance of each Asp residue (C_g_ atom) to the calcium ion during the MD simulation.


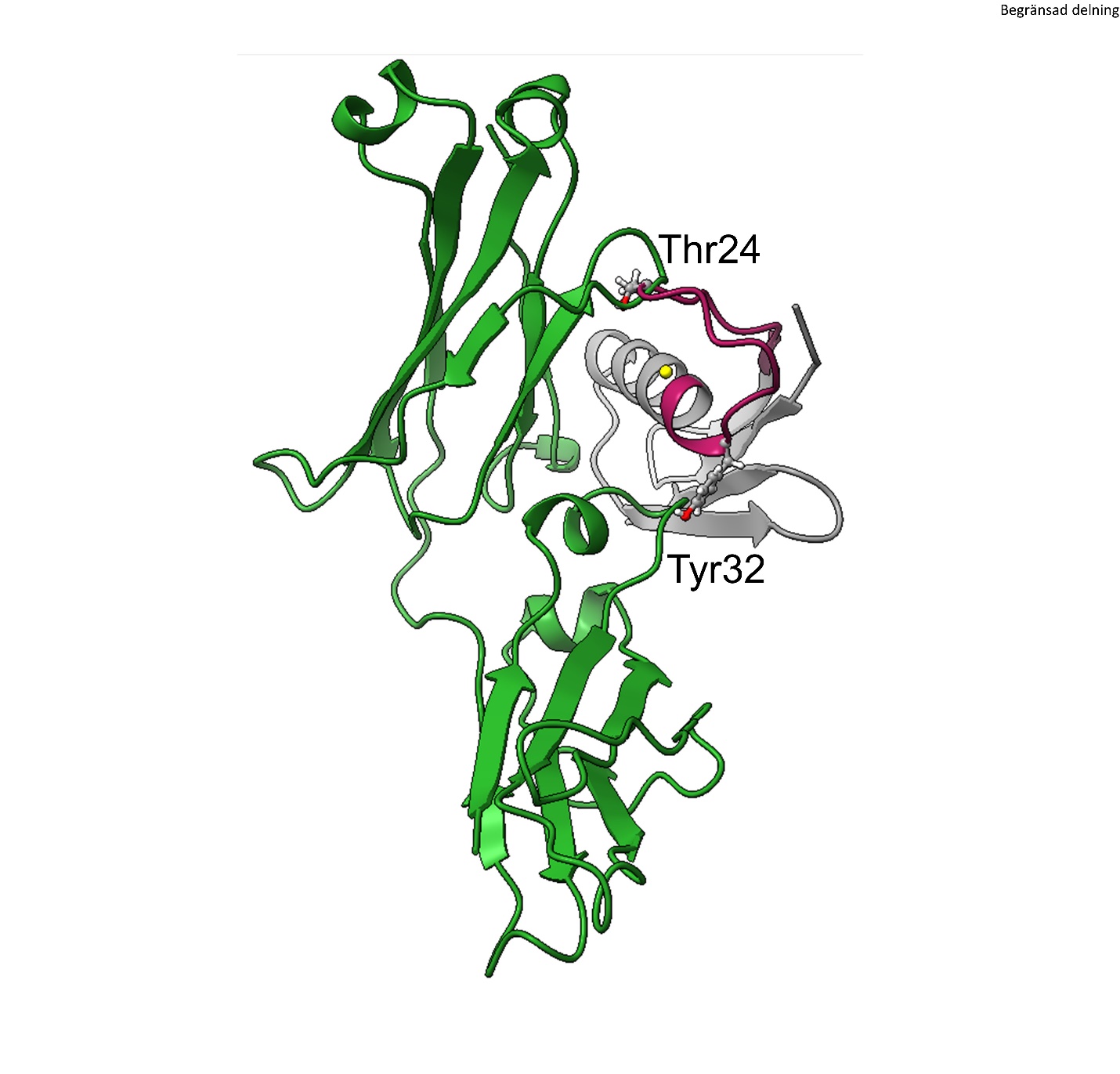


**Figure S14.** **Steric clashes hinder formation of a complex between C2_Ca V3_ and human Fc.** The model was created by superimposing the calcium loaded C2_Ca V3_ structure from MD with the C2:hFc complex (PDB: 1FCC). The calcium ion is coloured in yellow. In the superimposition, there are severe steric clashes between residues in the vicinity of Thr24 and Tyr32 of C2_Ca V3_.

**
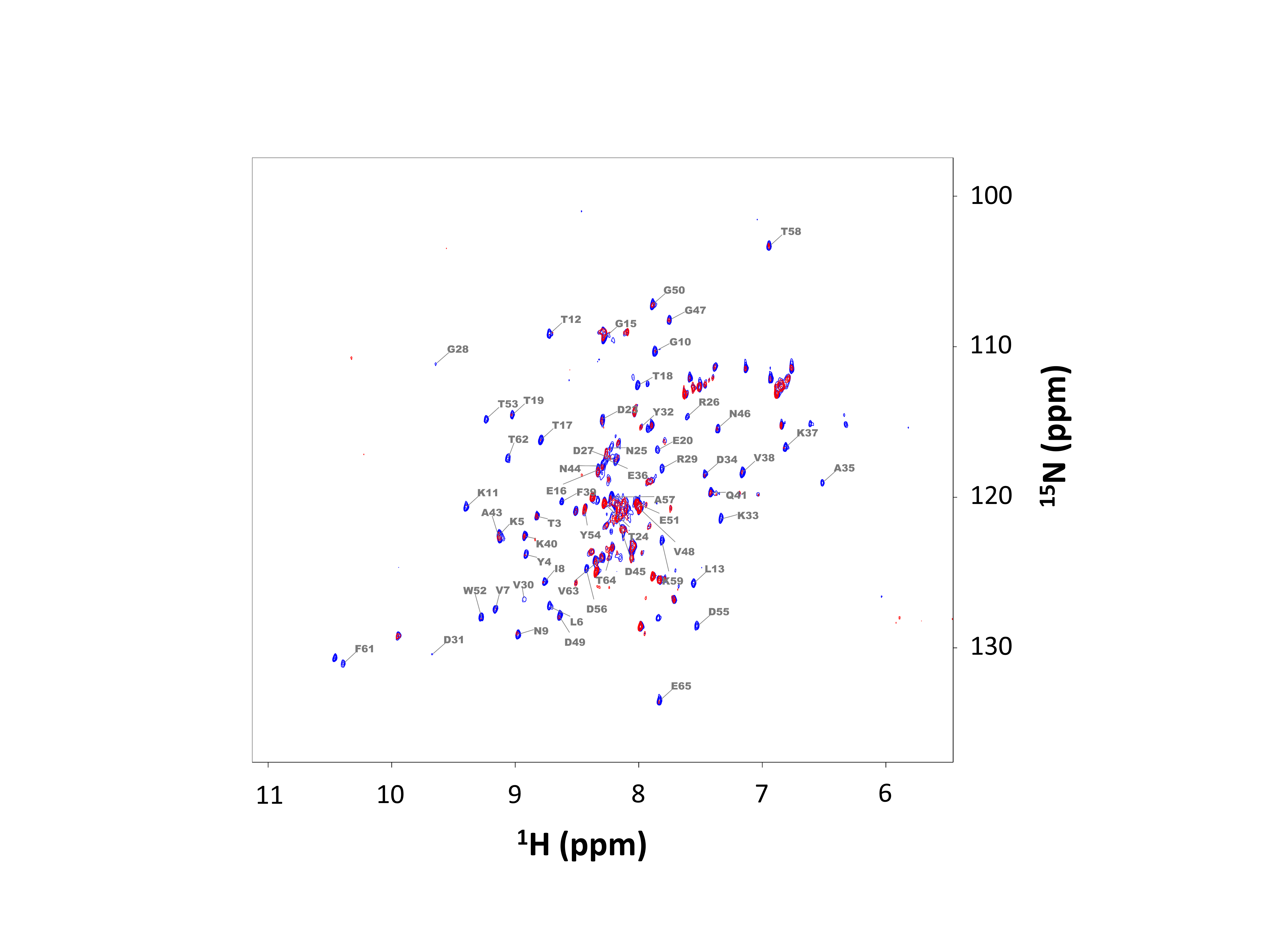
**

**Figure S15. Uniform broadening of C2_Ca V3_ resonances in complex with hFab and calcium.** Peak broadenings were observed in the ^1^H-^15^N HSQC correlation map of C2_Ca V3_ (blue) upon adding hFab (red) in a 1:10 molar ratio in the presence of 1 mM calcium. The signal attenuations continued with titration up to a 1:1 ratio, however, due to the intermediate exchange rate of the bound and free from, no new peak positions were detected. Data was collected on a 200 µM ^15^N-labeled C2_Ca V3_ sample in 20 mM MES at pH 6.0, 150 mM NaCl, 10% D_2_O and 1 mM CaCl_2_ at 298K.
